# Age-specific sex-differences in cerebral blood flow velocity in relation to haemoglobin levels

**DOI:** 10.1177/23969873241245631

**Published:** 2024-04-18

**Authors:** Sara Mazzucco, Linxin Li, Maria Assuncao Tuna, Peter M Rothwell

**Affiliations:** Wolfson Centre for Prevention of Stroke and Dementia, Nuffield Department of Clinical Neurosciences, University of Oxford, Oxford, UK

**Keywords:** Cerebral blood flow, sex, age, haemoglobin, transcranial Doppler

## Abstract

**Introduction:**

Cerebral blood flow (CBF) declines with age and abnormalities in CBF are associated with age-related cerebrovascular disease and neurodegeneration. Women have higher CBF than men, although this sex-difference diminishes to some extent with age in healthy subjects. The physiological drivers of these age/sex differences are uncertain, but might be secondary to age and sex-differences in haemoglobin (Hb) level. Hb levels are inversely correlated with CBF, are lower in women, and decline with age in men, but the interrelations between these factors have not been explored systematically either in healthy subjects or across the full age-range in patients with vascular risk factors. We aimed to determine the age-specific interrelations between sex, Hb, and CBF velocity in a large cohort of patients with cerebrovascular disease.

**Patients and methods:**

In patients with a recent transient ischaemic attack or minor stroke (Oxford Vascular Study) and no ipsilateral or contralateral stenosis of the carotid or intracranial arteries, we related peak-systolic velocity (PSV) and other parameters on transcranial Doppler ultrasound (TCD) of the middle cerebral artery to sex, age, Hb and vascular risk factors.

**Results:**

Of 958 eligible subjects (mean age/SD = 68.04/14.26, 53.2% male), younger women (age < 55 years) had higher CBF velocities than men (mean sex difference in PSV at age < 55 years = 16.31 cm/s; *p* < 0.001), but this difference declined with age (interaction *p* < 0.001), such that it was no longer significant at age 75–84 (∆PSV = 3.26 cm/s; *p* = 0.12) and was reversed at age ⩾ 85 (∆PSV = −7.42 cm/s; *p* = 0.05). These changes mirrored trends in levels of Hb, which were higher in men at age < 55 (∆Hb = 1.92 g/dL; *p* < 0.001), but steadily decreased with age in men but not in women (interaction *p* < 0.001), with no residual sex-difference at age ⩾ 85 (∆Hb = 0.12 g/dL; *p* = 0.70). There was an inverse correlation between Hb and PSV in both women and men (both *p* ⩽ 0.01), and the sex-difference in PSV at age < 55 was substantially diminished after adjustment for Hb (∆PSV = 6.92; *p* = 0.036; ∆PSV = 5.92, *p* = 0.13 with further adjustment for end-tidal CO_2_). In contrast, the sex difference in PSV was unaffected by adjustment for systolic and diastolic blood pressure, heart rate, and vascular risk factors (history of hypertension, diabetes, hyperlipidaemia and smoking).

**Discussion:**

CBF velocity is strongly correlated with Hb level at all ages, and sex-differences in CBF velocity appear to be explained in major part by age-related sex-differences in Hb.

## Graphical abstract

**Graphical abstract:**
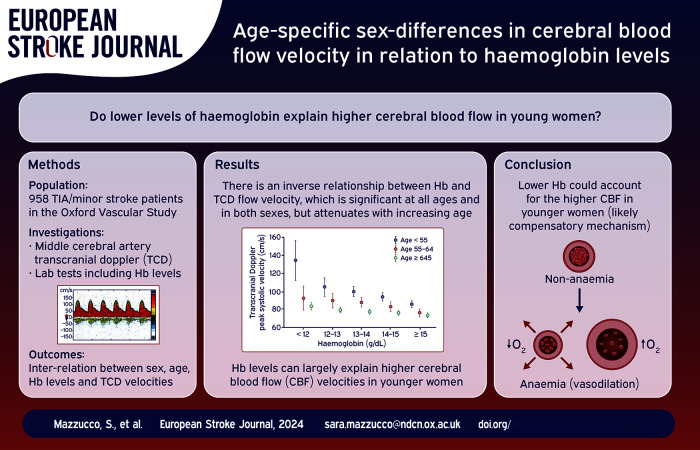

## Introduction

Regional cerebral blood flow (CBF) is tightly linked to metabolic demand, but there are relatively large inter-individual and group differences in global CBF.^1^ On average, global CBF is 15% higher in women versus men,^2–7^ particularly at younger ages, whether measured on Positron Emission Tomography,^3^ Xe^133^ Single-Photon Emission Tomography,^2^ arterial spin labelling magnetic resonance imaging (MRI),^4^ phase-contrast MRI^4^ or Transcranial Doppler sonography (TCD).^5–7^ The drivers of this sex-difference are still unclear, but investigators have tended to focus on brain-specific mechanisms in light of the tight neurovascular control of regional CBF. However, women have also been shown to have higher systemic blood flow than men after adjustment for blood-pressure and body-surface-area,^8^ suggesting that sex-differences in global CBF might simply reflect the systemic situation. Indeed, given the lower cortical synaptic density in women versus men,^3^ and no evidence of increased cerebral oxygen consumption in women, a higher brain-specific CBF requirement would be unexpected.^3^

Although CBF decreases with age in both sexes,^2–7,9^ the sex-difference in CBF could be age-dependent.^3,7^ However, human studies of CBF have usually been too small^2–7^ and too selective to reliably determine age and sex-specific norms, either because they have been explicitly limited to a particular age group,^7,9^ or have inadvertently excluded a high proportion of older individuals (e.g. excluding those with vascular risk factors^5,6^). There have also been no large studies of haemoglobin (Hb) levels as the potential explanation of the age-specific sex differences in CBF. That average Hb levels in younger women are about 15% lower than in men, and that this sex-difference decreases with age,^10^ suggests a potential link. Low Hb levels are associated with increased global CBF^11^ in animal models,^12^ children with sickle cell disease^13^ and adults with chronic renal failure,^14^ whereas polycythaemia is associated with reduced CBF.^15^ Transfusion^16^ and venesection^15^ are known to normalize CBF in each situation, respectively.^15^ Animal studies have shown that increased CBF in response to anaemia is due to a compensatory vasodilation of the small distal cerebral arteries in order to maintain oxygenation.^12^ There is also evidence that anaemia depletes cerebrovascular reserve, increasing the risk of stroke.^13^ These findings suggest that sex-differences in Hb could explain greater susceptibility of younger women than men to ischaemic injury when CBF is reduced, for example during cardiovascular surgery.^17–19^

We therefore aimed to explore the relation between age-specific sex differences in Hb levels and CBF in a cohort with a sufficient age-range to include both younger adults, in whom sex-differences in Hb and CBF are greatest, and the very elderly in whom these differences are lost. To facilitate recruitment of large numbers and to avoid exclusion biases due to contraindications (or disinclinations) to other imaging techniques, we used transcranial Doppler (TCD) to investigate CBF velocity. TCD is a non-invasive and inexpensive technique which has been widely used to investigate cerebral haemodynamics.^20^ While CBF is a volumetric concept, TCD measures blood flow velocity rather than volume. However, velocities in the MCA are correlated with blood flow volume in the internal carotid artery (ICA) measured through duplex ultrasound and phase-contrast MRI, with negligible changes in the proximal MCA diameter, even after pharmacologically-induced BP reductions.^4,20,21^

## Methods

This study was nested in the Oxford Vascular (OxVasc) Study, an ongoing population-based study of the incidence and outcome of all acute vascular events in a population of 92,728 individuals, irrespective of age, registered with 100 primary care physicians in nine practices in Oxfordshire, UK. Multiple methods of ascertainment are used for patients with TIA/stroke, as detailed elsewhere,^22^ including a daily, rapid-access TIA clinic, to which participating physicians and the local emergency department refer individuals with suspected TIA or non-disabling stroke (Supplemental Methods).

Patients are assessed by a study neurologist or stroke physician, who also provides their clinical care, and all presentations and investigations are reviewed by the senior study neurologist (PMR). Brain and vascular imaging to rule out extra/intra-cranial stenosis are obtained through 3T magnetic resonance imaging (MRI) with time-of-flight magnetic resonance angiography (MRA) of the intracranial vessels and a contrast-enhanced MRA of the large neck arteries, or brain computed tomography (CT) with contrast-enhanced CT angiography or Duplex ultrasound if MRI is contraindicated. Standard haematological parameters including Hb and haematocrit (Ht) are assessed at baseline, recorded from complete full blood count reports on blood samples obtained at the time of ascertainment.

All consenting patients with TIA/non-disabling stroke were eligible for inclusion in the current study if they had suitable temporal bone window. Patients with evidence of ⩾50% ICA or MCA stenosis on vascular imaging^23^ or with severe aortic valve disease were excluded from the analysis.

The OxVasc study was approved by the local ethics committee (OREC A: 05/Q1604/70) and consent was obtained from all participants.

### Transcranial Doppler

From November 1st 2011 all eligible subjects attending the OxVasc rapid-access TIA clinic underwent additional phenotyping (OxVasc Phenotyped Cohort), including TCD ultrasound. Patients were assessed at baseline and again at 1-month follow-up. TCD sonography (Doppler Box, Compumedics DWL, Singen, Germany) was performed by one of three experienced operators (SM, MT, LL). All TCD examinations were conducted in the same quiet dedicated room, with the patient awake, lying comfortably supine on a couch with a flat pillow, after 5 minutes of rest. Twenty minutes were allowed for the TCD scan; duration of each scan was recorded. Middle cerebral artery (MCA) blood flow velocities were recorded bilaterally with a handheld 2 MHz probe placed superior to the zygomatic arch, optimizing the depth that provided the best signal, usually 50 mm. TCD identification of the MCA is relatively easy in presence of an adequate bone window, with an acceptable angle of insonation.^24^ Each session was stored in the hard disc of the TCD device for subsequent off-line analysis of blood flow velocity including peak systolic velocity (PSV), end diastolic velocity (EDV) and mean flow velocity (MFV). Pulsatility index (PI = PVS-EDV/MFV) and measures of vascular resistance, including resistance index (RI = PSV-EDV/PSV) and cerebrovascular resistance index (CVRi = mean blood pressure/MFV) were also calculated. Lying blood pressure was measured in the left arm (A&D Medical, Tokyo, Japan) immediately before and after the scan. End-tidal CO_2_ (EtCO_2_) was monitored via nasal cannula (Capnocheck Plus; Smith Medical) throughout the procedure.^21^

All patients were invited to 1-month follow-up for repeated TCD assessment.

### Statistical analysis

Analyses included all eligible subjects recruited between November 1st 2011 and November 30th 2018. TCD parameters were given as the mean/SD of two measurements on each side. Measures of systolic blood pressure (SBP), diastolic blood pressure (DBP), mean blood pressure (MBP) and heart rate (HR) were given as the mean/SD of two measurements taken before and after the TCD.^22^ EtCO_2_ (KPa) was given as the average recorded throughout the TCD procedure; haemodynamic, cardiovascular and haematologic measures were compared by age and sex (defined as sex assigned at birth) using χ^2^ test or ANOVA as appropriate. Sensitivity analyses were run excluding current smokers, patients on antihypertensive treatment and women on hormone replacement therapy for menopause (HRT) treatment. The effect of the interaction between age and sex on each variable was assessed using age as a continuous variable. The correlation between haemodynamic variables and Hb was explored through lines of best fit on regression analysis in patients <55, 55–64 and ⩾65 years of age, reflecting pre-menopausal, peri-menopausal and post-menopausal age ranges.

To explore overall and within each age group the relative contribution of different parameters to each of the most relevant TCD variables, multiple regression analyses were carried out, with independent variables including sex, age, cardiovascular variables (SBP, DBP, HR), vascular risk factors (history of hypertension, diabetes, hyperlipidaemia, smoking) and EtCO_2_ (in the subgroup with this measure). To explore the effect of Hb levels on sex difference, models with and without Hb were developed to determine whether an attenuation of the correlation between sex and TCD measures was observed. The analysis was repeated excluding severely hypertensive patients (SBP ⩾ 160 mmHg) and 1 month after initial assessment in the subgroup of patients who underwent repeated TCD at 1-month follow-up. Statistical significance level was set to 0.05. All analyses were performed using SPSS version 27.

## Results

Of 1111 eligible subjects who underwent TCD screening at their first assessment, 115 were excluded due to ⩾50% intra/extra-cranial stenosis on vascular imaging and two due to severe aortic disease; 36 did not have a Hb measure, leaving 958 (86.4%) subjects for analysis (mean age/SD = 68.04/14.26, 509 male). Median time between symptom-onset and first assessment was 4 days (IQR 2,9); 67% of patients had a TIA and 21% had an acute ischaemic lesion on diffusion-weighted imaging. Median time from first to second assessment was 31 days (IQR 27,34) in the 786 (82.0%) subjects who had face-to-face follow-up. A subset of 690 (72.0%) patients had EtCO_2_ measures at their first assessment and 594 (75.6%) at second assessment. The demographic, clinical, haematological and TCD characteristics of these subgroups (Supplemental Table 1) were similar to those of the full cohort (Table 1).

**Table 1.** Demographic, clinical and physiological variables stratified by sex and age. Values are given as mean/SD unless otherwise specified.

	<55 years of age			55–64 years of age			65–74 years of age			75–84 years of age			⩾85 years of age		
	Males	Females	*p*	Males	Females	*p*	Males	Females	*p*	Males	Females	*p*	Males	Females	*p*
*N*	111	70		83	72		152	114		118	137		45	55	
Age	45.86/ 7.43	45.13/ 7.77	0.524	59.53/ 2.74	59.74/ 3.01	0.656	69.83/ 2.76	69.31/2.96	0.140	79.88/2.79	78.96/2.95	0.011	88.78/2.46	88.25/3.36	0.386
Haemoglobin (g/dL)	15.33/ 1.03	13.41/ 0.96	<0.001	14.69/ 1.45	13.53/ 1.30	<0.001	14.68/ 1.51	13.60/1.28	<0.001	13.92/1.49	13.19/1.24	<0.001	13.30/1.86	13.18/1.25	0.702
Haematocrit	0.45/ 0.03	0.40/ 0.03	<0.001	0.44/ 0.04	0.41/ 0.04	<0.001	0.44/ 0.04	0.41/0.04	<0.001	0.42/0.04	0.40/0.04	<0.001	0.40/0.05	0.40/0.04	0.492
Risk factors (%)															
Hypertension	28 (25)	17 (24)	0.887	37 (45)	26 (36)	0.284	95 (63)	62 (54)	0.183	74 (63)	87 (64)	0.896	30 (67)	38 (69)	0.796
Diabetes	13 (12)	2 (3)	0.035	16 (19)	10 (14)	0.371	22 (14)	10 (9)	0.157	19 (16)	17 (12)	0.398	8 (18)	3 (6)	0.050
Dyslipidaemia	21 (19)	10 (14)	0.405	34 (41)	20 (28)	0.086	60 (39)	33 (29)	0.075	59 (51)	55 (40)	0.098	19 (42)	17 (32)	0.299
Smoking history	66 (59)	39 (56)	0.698	43 (52)	30 (42)	0.237	105 (69)	45 (39)	<0.001	70 (59)	47 (34)	<0.001	25 (56)	23 (42)	0.171
Physiological variables															
Systolic blood pressure (mmHg)	135.90/ 17.87	133.45/ 16.88	0.350	138.80/ 19.56	136.62/ 19.36	0.488	144.82/ 19.84	148.29/20.64	0.167	146.35/17.57	154.53/23.75	0.002	151.87/21.17	158.75/22.07	0.118
Diastolic blood pressure (mmHg)	82.90/ 11.10	80.29/ 9.40	0.105	83.43/ 11.09	79.46/ 10.02	0.022	81.65/ 11.89	79.37/10.57	0.106	79.28/11.66	79.91/12.11	0.674	78.48/12.94	81.20/12.45	0.288
Mean blood pressure (mmHg)	100.38/ 12.61	97.76/ 10.75	0.153	101.63/ 12.82	98.27/ 11.87	0.096	102.47/ 13.32	101.91/12.86	0.731	101.60/12.16	104.54/14.31	0.083	102.70/13.32	106.91/13.52	0.122
Heart rate (bpm)	69.47/ 12.36	72.61/ 14.23	0.118	69.58/ 14.20	72.85/ 11.97	0.128	67.84/ 13.31	70.94/12.06	0.052	66.56/11.38	75.23/11.48	<0.001	67.11/12.79	72.64/11.74	0.027
Peak systolic velocity (cm/s)	87.05/ 15.21	103.36/ 18.33	<0.001	78.12/ 15.82	89.06/ 16.30	<0.001	76.49/ 15.06	82.16/15.52	0.003	73.68/16.95	76.94/16.04	0.117	78.38/18.19	70.96/19.26	0.052
End-diastolic velocity (cm/s)	41.22/ 8.00	47.96/ 9.29	<0.001	33.92/ 7.58	38.92/ 8.30	<0.001	30.21/ 7.39	31.49/7.44	0.167	26.37/6.77	26.74/6.34	0.658	24.41/7.56	23.27/6.33	0.412
Mean flow velocity (cm/s)	58.24/ 10.37	70.60/ 12.90	<0.001	50.02/ 10.80	59.09/ 11.33	<0.001	47.25/ 9.93	51.41/10.56	0.001	43.38/10.01	45.91/9.77	0.043	43.68/10.95	41.20/10.64	0.255
Pulsatility Index	0.79/ 0.14	0.79/ 0.12	0.870	0.90/ 0.17	0.86/ 0.12	0.109	0.99/ 0.21	1.00/0.17	0.926	1.10/0.23	1.10/0.18	0.964	1.26/0.26	1.16/0.23	0.043
Resistance index	0.53/ 0.05	0.54/0.05	0.265	0.57/ 0.06	0.56/ 0.05	0.625	0.60/ 0.07	0.62/0.06	0.095	0.64/0.08	0.65/0.06	0.146	0.69/0.07	0.67/0.07	0.120
Cerebrovascular resistance INDEX	1.78/ 0.39	1.43/0.31	<0.001	2.15/ 0.68	1.74/ 0.45	<0.001	2.27/ 0.59	2.09/0.59	0.010	2.48/0.71	2.40/0.70	0.371	2.51/0.75	2.79/0.87	0.094

**Figure 1.**
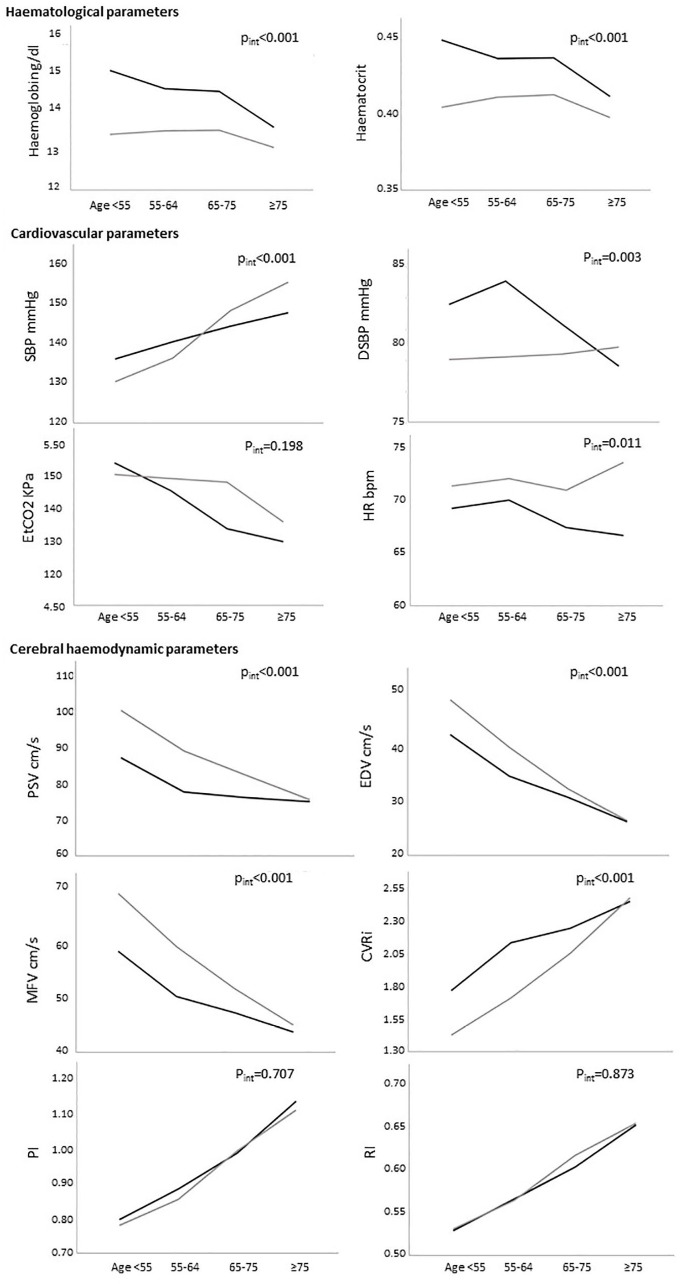
Mean haematological, cardiovascular, and cerebral haemodynamic variables by age groups. Dark line: men; light line: women; SBP: systolic blood pressure; DBP: diastolic blood pressure; HR: heart rate; EtCO_2_: end-tidal CO_2_; Ht: haematocrit; Hb: haemoglobin; PSV: peak systolic velocity; EDV: end diastolic velocity; MFV: mean flow velocity; CVRi: cerebrovascular resistance index; PI: pulsatility index; RI: resistance index. The *p*-values in the figure refer to sex/age interaction.

Mean/SD SBP increased with age in both sexes (Table 1 and Figure 1), but particularly in women (133.45/16.88 mmHg at age < 55 vs 155.75/23.30 mmHg at age ⩾ 75; *p* < 0.001) compared with men (135.95/17.87 mmHg vs 147.87/18.73 mmHg; *p* < 0.001), with a similar trend in subjects on no antihypertensive medication at the time of assessment (Supplemental Table 2). EtCO_2_ decreased with age in both sexes, and was marginally higher in women than in men (Figure 1 and Supplemental Table 1).

### Age and sex differences in Hb and TCD variables

Hb levels were lower in women than in men at all ages (Figure 1), but this sex-difference decreased with age (∆Hb = 1.92 g/dL at age < 55, *p* < 0.001 and 0.12 g/dL at age ⩾ 85, *p* = 0.70), due mainly to a fall in Hb levels with age in men, but relative stability in women up to age 75. The same trends were seen after excluding women on HRT (Supplemental Table 3), current smokers (Supplemental Table 4) and patients on antihypertensive medications (Supplemental Table 2).

The age/sex-dependent trends in CBF measures on TCD mirrored those in Hb (Figure 1), with younger women displaying higher CBF velocities. Although PSV, EDV and MFV fell with increasing age in both men and women, the difference in PSV between men and women decreased with age (interaction *p* < 0.001), was no longer significant at age 75–84 (∆PSV = 3.26 cm/s; *p* = 0.12) and was reversed at age ⩾ 85 (∆PSV = −7.42 cm/s; *p* = 0.05). The same trend was observed for EDV, MFV and CVRi, whereas PI and RI did not differ between men and women at any age.

### Correlation between TCD velocities and Hb

**Figure 2.**
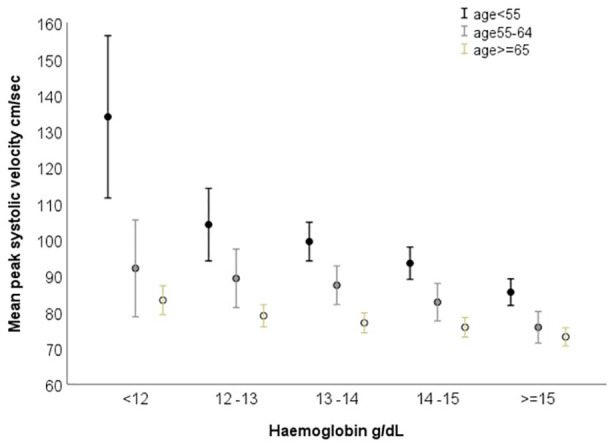
Mean peak systolic velocity by level of haemoglobin and age.

On univariate analysis there was an inverse correlation between Hb and TCD blood flow velocities, particularly PSV, but also EDV, MFV, RI and CVRi, which was significant across ages, but became flatter with increasing age (Figure 2 and Supplemental Table 5). This inverse correlation between Hb and TCD blood flow velocities was present independently in both men and women (Supplemental Figure 1). We explored the possibility of a non-linear relationship between PSV and Hb, and the model that best fitted the data was cubic (*R*^2^cubic = 0.237 at age ⩽ 55, *p* < 0.001; *R*^2^cubic = 0.138 at age > 55 and ⩽ 65, *p* < 0.001; R^2^cubic = 0.042 at age ⩾ 65, p < 0.001; Supplemental Figure 2).

### Multi-regression analysis

On multi-regression analysis of the determinants of PSV, the age-specific sex-differences in PSV remained after adjusting for age, SBP, DBP, HR, history of diabetes, hypertension, hyperlipidaemia, smoking habit and EtCO_2_ (Table 2 and Supplementary Table 6). However, the sex-differences in PSV were significantly attenuated after further adjustment for Hb, particularly at age < 55 years (unadjusted ∆PSV = 15.9 cm/s; *p* < 0.001 vs fully adjusted ∆PSV = 6.9; *p* = 0.036; ∆PSV = 5.9, *p* = 0.13 with further adjustment for end-tidal CO_2_). A similar trend was confirmed in analyses excluding severely hypertensive patients and repeated 1 month after initial assessment in the subgroup of 786 patients who underwent repeated TCD at 1-month follow-up (Supplemental Table 7). Supplemental Tables 8 and 9 show similar trends for EDV and MFV.

**Table 2.** Multivariate regression analysis performed in the whole cohort and in the group of patients with end-tidal CO_2_ measures, using peak systolic velocity as dependent variable, and sex (female vs male), age, systolic and diastolic blood pressure, heart rate, history of hypertension, diabetes, hyperlipidaemia and smoking habit as independent variables (Model A). Model B included haemoglobin as one of the independent variables, to explore its effect on the correlation between sex and peak systolic velocity. Model C and D included end-tidal CO_2_ (C) and end-tidal CO_2_ and haemoglobin (D) as independent variables in the group of patients with end-tidal CO_2_ measures.

	All patients				Patients with end-tidal CO_2_			
	Model A	*p*	Model B	*p*	Model C	*p*	Model D	*p*
All patients								
*B* (95% CI)	3.71 (1.56,5.87)	<0.001	0.88 (–1.37,3.13)	0.443	3.37 (0.83,5.91)	0.009	0.12 (–2.51,2.75)	0.927
Adjusted *R*^2^	0.254		0.290		0.291		0.336	
Number	958		958		690		690	
Age < 55 years								
*B* (95% CI)	15.89 (10.97,20.80)	<0.001	6.92 (0.45,13.40)	0.036	16.33 (10.43,22.22)	<0.001	5.92 (–1.81,13.65)	0.132
Adjusted *R*^2^	0.288		0.346		0.300		0.371	
Number	181		181		137		137	
Age 55–64 years								
B (95% CI)	7.96 (2.63,13.29)	0.004	5.10 (–0.54,10.74)	0.076	8.18 (1.63,14.74)	0.015	5.01 (–1.90,11.92)	0.153
Adjusted R^2^	0.134		0.169		0.193		0.231	
Number	155		155		110		110	
Age ⩾ 65 years								
*B* (95% CI)	–0.64 (–3.30,2.03)	0.638	–2.31 (–5.03,0.41)	0.096	–2.21 (–5.32,0.90)	0.163	–4.02 (–7.17,–0.86)	0.013
Adjusted *R*^2^	0.166		0.191		0.199		0.231	
Number	622		622		443		443	

## Discussion

Our findings suggest that age-specific sex differences in Hb levels account for most of the age-specific differences in CBF velocity between men and women and could provide an explanation for the large sex differences in CBF at younger ages, where the difference in Hb is greatest.

We found an inverse relationship between Hb and CBF velocity, which is significant at all ages and in both sexes, but attenuates with increasing age, which may be due to the age-related decreases in cerebral arterial compliance and compensatory dilatation in response to low Hb levels. Studies in animals^12^ and in young people with anaemia due to sickle cell disease^13^ or in patients with chronic renal failure,^14^ showed that normal O_2_ delivery is preserved via a compensatory increase in CBF through vasodilation in small distal arteries. This study suggests that this compensatory mechanism could be operating not only in patients with severe anaemia, but also at the lower end of the normal ranges of Hb in younger women.

Our study has some weaknesses. Firstly, we have not directly measured blood viscosity and oxygen delivery in our patients. Higher CBF with low Ht and Hb could simply be due to reduced viscosity rather than to a compensatory response to reduced oxygen delivery. However, that the inverse correlation between CBF and Ht/Hb is mediated by blood oxygen content rather than by viscosity was convincingly proven by Brown and Marshall’s^15^ work on anaemia, polycythaemia and paraproteinaemia. Moreover, a viscosity-mediated effect would probably not vary with age. Secondly, we had very limited data on the hormonal state of women in our study. Oestrogen levels during the menstrual cycle correlate with blood flow in the internal carotid artery,^25^ and are therefore likely to contribute to higher CBF in women at younger age. However, the sex-difference in CBF extends beyond the average age for menopause, even when excluding the group of women on hormone replacement therapy. Thirdly, our study measures blood flow velocities rather than volume, and cannot correct for differences in arterial diameter between men and women. However, a sex-difference in CBF has been observed with methods independent from arterial diameter,^3,11^ and was confirmed when normalizing CBF by brain tissue mass.^4^ Fouthly, our study included patients with vascular risk factors rather than healthy volounteers and was integrated into a clinical service. However, TCD and blood pressure assessments were made in a controlled environment by experienced neurosonologists, and although the patients had had a recent TIA or minor stroke, the event was usually 4 days prior to assessment and any neurological deficit had completely resolved prior to assessment in the majority. Results of the analysis repeated 1 month after the initial assessment, when any cerebral dysregulation is likely to have resolved, were similar to those of the main analysis. Although the majority of patients had vascular risk factors, they had vascular imaging to rule out occult stenosis. Finally, we did not perform vasomotor reactivity studies to test the extent of depleted reserve at low Hb levels. However, reduced vasomotor reserve has been documented previously in women versus men, particularly at younger ages,^7^ although it hasn’t to our knowledge been related to haemoglobin levels. Moreover, we estimated cerebrovascular resistance through CVRi, a ratio of mean blood pressure to CBF.^26^ As expected, as well as PI and RI, CVRi increased with age in both sexes, but was lower in females than in males up to the age of 75, with the difference between men and women decreasing with age such that it was no longer significant at age 75–84 and was reversed at age ⩾ 85. Higher CBF in the face of low cerebrovascular resistance suggests vasodilation with reduced residual dilatory reserve, in line with our hypothesis.^12^

The real-world nature of our study cohort has the advantage of comparability with some of the clinical groups for whom our findings have implications. For example, among patients undergoing major cardiovascular surgey, where younger women have worse outcomes than men, with higher mortality due to stroke,^17–19,27^ compensatory vasodilation due to anaemia in younger women could lead to depleted vasomotor reserve and increased risk of stroke.^13^ Indeed, the same may be true of all major surgery where high blood loss can be expected.^28^ It may be possible that other conditions associated with a reduction in CBF, such as migraine or reversible cerebral vasoconstriction syndrome, could impact predominantly younger women due to already depleted vasomotor reserve. Our study cohort is also appropriate for consideration of the possible interactions between CBF, age and Hb and the mechanisms underlying stroke and vascular dementia,^29^ and possibly of neurodegenerative disease more widely given the role of vascular risk factors in the aetiology of Alzheimer dementia and motor neurone disease.^30,31^

Given that low haemoglobin levels in young women can be largely corrected with iron supplementation,^32^ a better understanding of the impact on CBF could inform global health policies on screening and treatment of anaemia, particularly in young women and children in low and middle income countries.^33,34^ Lastly, the relationships between age, sex, CBF and Hb need to be taken into account in research, particularly on sex differences in disease risks and outcomes.

In conclusion, given the well established causal link between Hb and CBF in various disease states, our study shows that the age-dependent sex-difference in Hb could largly account for higher levels of CBF in women, particularly at younger ages, where it is likely to be a compensatory mechanism to maintain normal O_2_ delivery.

## Supplemental Material

sj-docx-1-eso-10.1177_23969873241245631 – Supplemental material for Age-specific sex-differences in cerebral blood flow velocity in relation to haemoglobin levels Supplemental material, sj-docx-1-eso-10.1177_23969873241245631 for Age-specific sex-differences in cerebral blood flow velocity in relation to haemoglobin levels by Sara Mazzucco, Linxin Li, Maria Assuncao Tuna and Peter M Rothwell in European Stroke Journal
