# Supplementary material for: Age-specific sex-differences in cerebral blood flow velocity in relation to haemoglobin levels
Source: Eur Stroke J. 2024 Apr 18;9(3):772–80. doi: 10.1177/23969873241245631 (PMC11343687; doi:10.1177/23969873241245631)
Supplement: sj-docx-1-eso-10.1177_23969873241245631 – Supplemental material for Age-specific sex-differences in cerebral blood flow velocity in relation to haemoglobin levels [file sj-docx-1-eso-10.1177_23969873241245631.docx]

**SUPPLEMENTARY MATERIAL**

**Age-specific sex-differences in cerebral blood flow velocity in relation to haemoglobin levels**

Sara Mazzucco MD, PhD, Linxin Li MD, DPhil, Maria Assuncao Tuna MD, DPhil, Peter M Rothwell MD, PhD, FmedSci, on behalf of the Oxford Vascular Study Phenotyped cohort

Wolfson Centre for Prevention of Stroke and Dementia, Nuffield Department of Clinical Neurosciences, University of Oxford

**Index:**

**Supplementary methods**…………………………………………………………………Page 2

OxVasc Methodology……………………………………………………………………....Page 2

Transcranial Doppler variables……………………………………………………………Page 4

Transcranial Doppler inter-observer agreement..……………………………………….Page 5

**Supplementary results**…………………………………………………………………...Page 7

Supplementary Table 1…………………………………………………………………….Page 8

Supplementary Table 2…………………………………………………………………….Page 9

Supplementary Table 3…………………………………………………………………….Page 10

Supplementary Table 4…………………………………………………………………….Page 11

Supplementary Table 5…………………………………………………………………….Page 13

Supplementary Table 6A….……………………………………………………………….Page 14

Supplementary Table 6B….……………………………………………………………….Page 15

Supplementary Table 6C….……………………………………………………………….Page 16

Supplementary Table 6D….……………………………………………………………….Page 17

Supplementary Table 7….…………………………………………………………………Page 18

Supplementary Table 8….…………………………………………………………………Page 19

Supplementary Table 9….…………………………………………………………………Page 20

Supplementary Figure 1A….………………………………………………………………Page 21

Supplementary Figure 1B….………………………………………………………………Page 22

Supplementary Figure 2….……………………………………………………………......Page 23

**Detailed bibliography**……………………………………………………………………..Page 24

**Supplementary methods**

1. **OXVASC methodology**

**Study population**

The Oxford Vascular Study (OXVASC) is a prospective, population-based cohort study of all incident acute vascular events in all territories (transient ischaemic attack, stroke, acute coronary and peripheral vascular events). During the period of the current sub study, the OXVASC study population consisted of all 92,728 individuals, irrespective of age, registered with 100 general practitioners (GPs) in nine general practices in Oxfordshire, UK. In the UK, general practices provide primary health care for registered individuals and hold a lifelong record of all medical consultations (from the National Health Service [NHS] and private health care), and details of treatments, blood pressure, and investigations. In Oxfordshire, an estimated 97% of the true residential population is registered with a general practice, with most non-registered individuals being young students. All participating practices held accurate age-sex patient registers, and allowed regular searches of their computerised diagnostic coding systems. The practices had all collaborated on a previous population-based study, for which they were originally selected to be representative of the urban and rural mix and the deprivation range of Oxfordshire as a whole. Based on the index of multiple deprivation (IMD), the population was less deprived than the rest of England, but had a broad range of deprivation.

The OXVASC population is 94% white people, 3% Asian, 2% Chinese, and 1% Afro-Caribbean. The proportion of whites is similar to that of the UK as a whole (88% white) and to many other western countries (Australia - 90%; France - 91%; Germany - 93.9%).

**Case ascertainment**

After a 3-month pilot study, the study started on April 1, 2002, and is ongoing. Ascertainment combined prospective daily searches for acute events (hot pursuit) and retrospective searches of hospital-care and primary-care administrative and diagnostic coding data (cold pursuit).

Hot pursuit was based on:

1. A daily (weekdays only), urgent open-access “TIA clinic” to which participating general practitioners (GPs) and the local accident and emergency department (A&E) send all individuals with suspected TIA or stroke whom they would not normally admit to hospital, with alternative on-call review provision at weekends. Patients too frail to attend are assessed at their residence by a study nurse or doctor. Patients are assessed at initial face-to-face interview and clinical data are cross-checked with primary care records. Data are collected on demographics and vascular risk factors including male sex, history of hypertension, diabetes mellitus, hypercholesterolemia, smoking habit (ex or current smoker), atrial fibrillation, and ongoing medications.
2. Daily searches and case note review of admissions to the Emergency Assessment Unit, Medical Short Stay Unit, Coronary Care Unit and Cardiothoracic Critical Care Unit, Cardiology, Cardiothoracic, and Vascular Surgery wards, Acute Stroke Unit, Neurology ward and all other general wards when indicated.
3. Daily searches of the local A&E and eye hospital attendance registers.
4. Daily identification via the Bereavement Office of patients dead on arrival at hospital or who died soon after.
5. Daily searches of lists of all patients from the study population in whom a troponin-I level had been requested.
6. Daily assessment of all patients undergoing diagnostic coronary, carotid and peripheral angiography, angioplasty, stenting or vascular surgical procedures in any territory to identify both total burden of vascular invention and any potential missed prior acute events.

Cold pursuit procedures were:

1. Frequent visits to the study practices and monthly searches of practice diagnostic codes.
2. Monthly practice-specific list of all patients admitted to all acute and community NHS hospitals.
3. Monthly listings of all referrals for brain or carotid imaging studies performed in local hospitals.
4. Monthly reviews of all death certificates and coroners reports to review out-of-hospital deaths.
5. Practice-specific listings of all ICD-10 death codes from the local Department of Public Health.

Patients found on GP practice searches who have an event whilst temporarily out of Oxfordshire are included, but visitors who were not registered with one of the study practices are excluded. A study clinician assessed patients as soon as possible after the event in the hospital or at home. Informed consent was sought, if possible, or assent was obtained from a relative. Data are collected using event-specific forms, for TIA and stroke, acute coronary syndrome or acute peripheral vascular events. Standardised clinical history and cardiovascular examination are recorded. Information recorded from the patient, their hospital records and their general practice records includes details of the clinical event, vascular risk factors, medication, past medical history, all investigations relevant to their admission (including blood results, electrocardiography, brain imaging and vascular imaging-duplex ultrasonography, CT-angiography, MR-angiography or DSA) and all interventions occurring subsequent to the event.

If a patient died before assessment, we obtained an eyewitness account of the clinical event and reviewed any relevant records. If death occurred outside the hospital or before investigation, the autopsy result was reviewed. Clinical details are sought from primary care physicians or other clinicians on all deaths of possible vascular aetiology.

All surviving TIA and stroke patients are followed-up face-to-face at 1, 6, 12, 60 and 120 months after the initial event by a research nurse or physician and all recurrent vascular events were recorded together with the relevant clinical details and investigations. If face-to-face follow up is not possible, telephone follow-up is performed or enabled via the general practitioner. All recurrent vascular events that presented to medical attention would also be identified acutely by ongoing daily case ascertainment within OXVASC. If a recurrent vascular event was suspected at a follow-up visit or referred by the GPs to clinic or admitted, the patient was re-assessed and investigated by a study physician.

**Definitions of events**

Although new definitions for stroke and TIA have been suggested, in order to enable comparison with previous studies, the classic definitions of TIA and stroke are used throughout. A stroke is defined as rapidly developing clinical symptoms and/or signs of focal, and at time global (applied to patients in deep coma and to those with subarachnoid haemorrhage), loss of brain function, with symptoms lasting more than 24 hours or leading to death, with no apparent cause other than that of vascular origin. A TIA is an acute loss of focal brain or monocular function with symptoms lasting less than 24 hours and which is thought to be caused by inadequate cerebral or ocular blood supply as a result of arterial thrombosis, low flow or embolism associated with arterial, cardiac or haematological disease. A ‘minor stroke’ is defined as a stroke with NIHSS score of ≤3. All diagnoses were reviewed by a senior neurologist (PMR). With the high rate (97%) of imaging or autopsy in OXVASC, strokes of unknown type were coded as ischaemic.

**Transcranial Doppler variables**

**Peak Systolic Velocity (PSV)** measures the greatest magnitude of Doppler shift. PSV measured in the middle cerebral artery of foetuses between 18-40 weeks of gestation is strongly correlated with anaemia. It is a predictor of moderate or severe anaemia, and is used as a validated marker of degree of foetal anaemia. *(Mari G. Noninvasive diagnosis by Doppler ultrasonography of foetal anaemia due to maternal red-cell alloimunization. New Engl J Med 2000;342:9-14)*

**End-diastolic Velocity (EDV)** corresponds to the point of the Doppler spectrum at the end of the cardiac cycle, just before the systolic peak. Organs like the brain or the kidney, requiring positive perfusion throughout the entire cardiac cycle, typically exhibit low resistance flow. This is characterised by high diastolic flow and positive EDV. In contrast, high resistance vessels are characterised by low or negative EDV. Therefore EDV is strongly related to peripheral resistance. It has been shown that EDV reliably reflects changes in perfusion and reperfusion after thrombolysis, with even a small increase in early post-recanalisation EDV being associated with significant neurological and functional improvement. This suggests that EDV is a clinically relevant marker of cerebral perfusion. (*Alexandrov AV et al. End-diastolic velocity increase predicts recanalization and neurological improvement in patients with ischemic stroke with proximal arterial occlusions receiving reperfusion therapies. Stroke 2010; 41: 948–952. 24. Mazzucco S at al. Hemodynamic correlates of transient cognitive impairment after transient ischemic attack and minor stroke: A transcranial Doppler study. Int J Stroke 2016; 11: 978–986.)*

**Mean Flow Velocty (MFV)** is nowadays automatically calculated by ultrasound machines, but can be manually calculated as equal to (PSV + (EDV *2))/3. It is widely used as the main TCD measure in intra-operative monitoring or for research purposes. Time-averaged maximum mean flow velocity (TAMMV) is also used to stratify the risk of stroke in children with sickle cell disease. *(Adams RJ, et al. Prevention of a first stroke by transfusions in children with sickle cell anemia and abnormal results on transcranial Doppler ultrasonography. N Engl J Med 1998; 339: 5–11.)*

**Pulsatility index** (**PI** = PVS-EDV/MFV) and **Resistance index** (**RI** = PSV-EDV/PSV) provide a measure of downstream vascular resistance. They are positively correlated with resistance in distal smaller vessels, increasing with distal occlusion/constriction, and decreasing with peripheral vasodilation. *(Aaslid R,Transcranial Doppler. Raven Press, Ltd., New York,1992)*

**Cerebrovascular resistance index** (**CVRi**= mean blood pressure/MFV) can be calculated from the ratio of cerebral perfusion pressure to cerebral blood flow, where cerebral perfusion pressure is the difference between mean arterial pressure and intracranial pressure. Under conditions where intracranial pressure is normal, CVRi can be estimated from the ratio of mean arterial pressure, reflecting the relationship between blood pressure and cerebral blood flow. (*Markus HS. Cerebrovascular abnormalities in Alzheimer’s dementia: a more tractable treatment target? Brain 2017; 140: 1822–1825.)*

**Transcranial Doppler inter-observer agreement:**

Transcranial Doppler (TCD) scans were performed by three certified Neurosonologists (SM, LL, MT), on average with more than 6 years of TCD experience. SM provided supervision and quality assurance for TCD scans throughout the study; SM is part of the certification board of the Italian Society of Neurosonology and Cerebral Haemodynamic, and is the ambassador for the UK of the European Society of Neurosonology and Cerebral Haemodynamic, with over 20 years of TCD experience.

The OxVasc TCD methodology has been previously published (*Mazzucco S, on behalf of the Oxford Vascular Study. International Journal of Stroke 2016;11:978-986; Mazzucco S, Oxford Vascular Study Phenotyped Cohort. Lancet Neurology 2018;17:609-617; Mazzucco Set al. JAMA Neurology. 2020;77:1-9; Mazzucco S, on behalf of the Oxford Vascular Study Phenotyped cohort. European Stroke Journal. 2021;6:245-253; Mazzucco S, et al. International Journal of Stroke 2022;17:1114-1120*)

Eighty-five percent of scans were performed by SM and LL; agreement between these two operators is as follows:

Linear regression for Mean Flow Velocity (MFV)


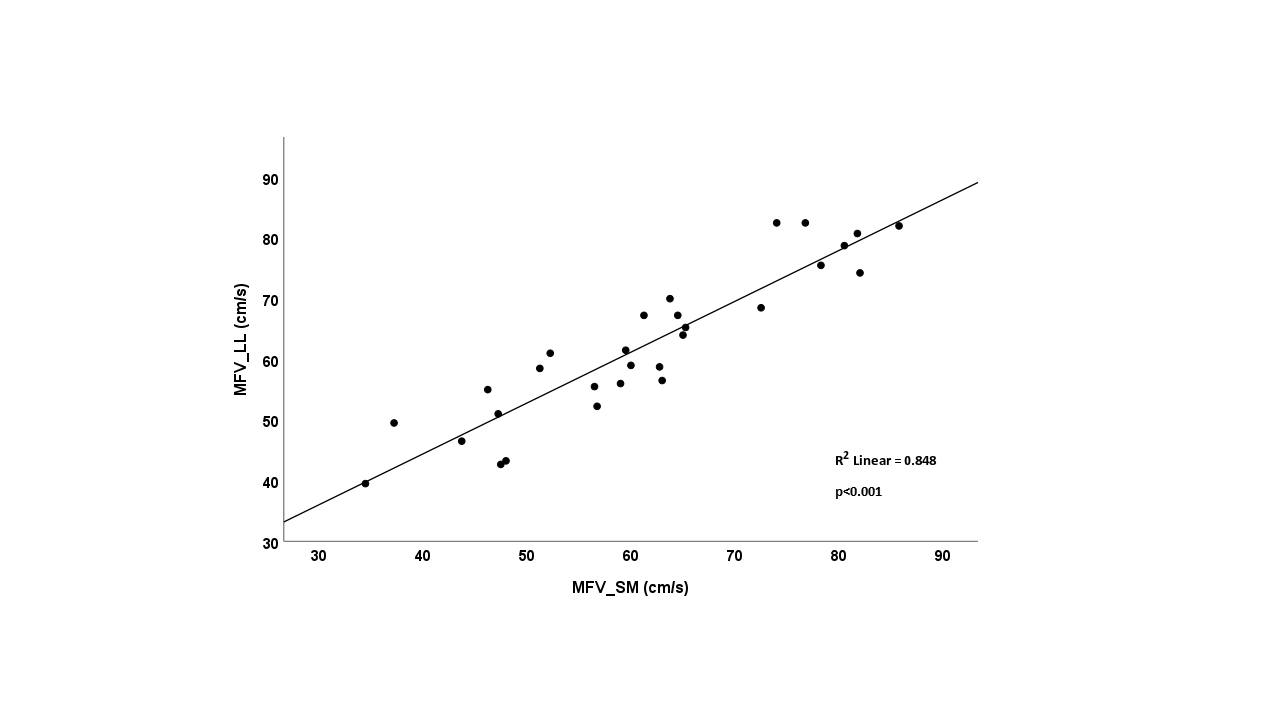


Bland–Altman plot for MFV:


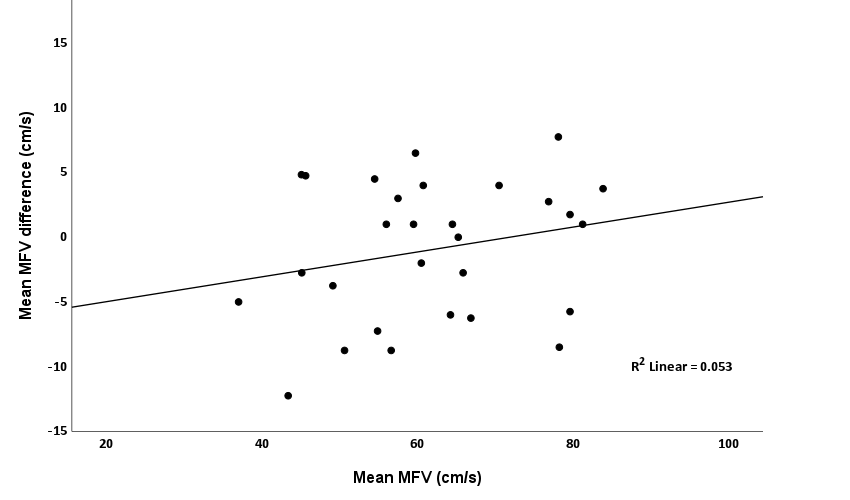


**Supplementary Results**

|  | **<55 years of age** | | | **55-64 years of age** | | | **65-74 years of age** | | | **75-84 years of age** | | | **≥85 years of age** | | |
| --- | --- | --- | --- | --- | --- | --- | --- | --- | --- | --- | --- | --- | --- | --- | --- |
|  | **Males** | **Females** | ***p*** | **Males** | **Females** | ***p*** | **Males** | **Females** | ***p*** | **Males** | **Females** | ***p*** | **Males** | **Females** | ***p*** |
| N | 83 | 54 |  | 61 | 49 |  | 110 | 84 |  | 81 | 98 |  | 33 | 36 |  |
| Age | 44.80/  7.95 | 44.96/  8.10 | 0.905 | 59.43/  2.80 | 59.90/  3.16 | 0.408 | 69.86/  2.72 | 69.45/  2.85 | 0.308 | 80.01/  2.77 | 79.05/  3.01 | 0.029 | 88.79/  2.50 | 87.72/  2.67 | 0.092 |
| Haemoglobin (g/dL) | 15.33/  1.08 | 13.30/  0.91 | <0.001 | 14.69/  1.52 | 13.56/  1.36 | <0.001 | 14.70/  1.51 | 13.73/  1.21 | <0.001 | 13.99/  1.46 | 13.21/  1.27 | <0.001 | 13.42/  1.73 | 13.33/  1.21 | 0.789 |
| Haematocrit | 0.45/  0.03 | 0.40/  0.02 | <0.001 | 0.43/  0.04 | 0.41/  0.04 | <0.001 | 0.44/  0.04 | 0.41/  0.04 | <0.001 | 0.42/  0.04 | 0.40/  0.04 | 0.001 | 0.41/  0.05 | 0.40/  0.04 | 0.676 |
| End-tidal CO_2_ (KPa) | 5.36/  0.54 | 5.19/  0.57 | 0.075 | 5.07/  0.52 | 5.32/  0.66 | 0.027 | 4.99/  0.59 | 5.20/  0.64 | 0.022 | 4.92/  0.63 | 5.02/  0.55 | 0.258 | 4.67/  0.69 | 4.88/  0.69 | 0.228 |
| Systolic blood pressure (mmHg) | 134.60/  17.09 | 132.88/  17.90 | 0.574 | 140.07/  20.99 | 135.74/  17.82 | 0.253 | 144.41/  19.44 | 147.28/  19.94 | 0.315 | 144.04/  16.56 | 153.97/  21.26 | <0.001 | 148.91/  18.72 | 155.92/  20.82 | 0.147 |
| Diastolic blood pressure (mmHg) | 82.39/  10.93 | 79.46/  10.14 | 0.118 | 84.02/  12.22 | 79.63/  10.57 | 0.049 | 81.81/  11.88 | 79.00/  10.27 | 0.085 | 78.59/  11.85 | 79.75/  12.30 | 0.525 | 79.14/  12.82 | 81.06/  11.93 | 0.522 |
| Mean blood pressure (mmHg) | 99.51/  12.17 | 96.94/  11.54 | 0.221 | 102.38/  14.11 | 98.00/  11.67 | 0.085 | 102.37/  13.15 | 101.46/  12.24 | 0.627 | 100.36/  11.67 | 104.19/  13.62 | 0.049 | 102.09/  12.47 | 105.65/  13.33 | 0.257 |
| Heart rate (bpm) | 69.36/  12.32 | 71.43/  14.46 | 0.373 | 69.53/  14.71 | 71.66/  10.65 | 0.397 | 66.88/  12.85 | 70.23/  11.81 | 0.064 | 66.08/  11.82 | 74.36/  11.38 | <0.001 | 66.32/  12.42 | 69.67/  9.88 | 0.218 |
| Peak systolic velocity (cm/s) | 87.80/  15.33 | 104.01/  19.37 | <0.001 | 78.08/  16.84 | 90.34/  16.13 | <0.001 | 76.20/  14.54 | 81.69/  14.96 | 0.011 | 74.64/  17.26 | 77.38/  15.19 | 0.262 | 76.26/  18.46 | 68.72/  18.62 | 0.096 |
| End-diastolic velocity (cm/s) | 42.06/  7.97 | 47.63/  9.49 | <0.001 | 33.89/  7.78 | 50.01/  8.61 | <0.001 | 30.27/  6.93 | 31.41/  6.97 | 0.159 | 26.76/  6.92 | 27.01/  6.02 | 0.798 | 24.15/  7.31 | 23.22/  6.72 | 0.581 |
| Mean flow velocity (cm/s) | 59.11/  10.51 | 70.54/  13.43 | <0.001 | 50.03/  11.30 | 60.42/  11.40 | <0.001 | 47.03/  9.34 | 50.95/  9.92 | 0.005 | 43.93/  10.11 | 47.13/  9.28 | 0.131 | 42.70/  10.78 | 40.08/  11.06 | 0.323 |
| Pulsatility Index | 0.78/  0.13 | 0.80/  0.13 | 0.287 | 0.89/  0.18 | 0.84/  0.12 | 0.087 | 0.99/  0.21 | 1.00/  0.16 | 0.856 | 1.10/  0.25 | 1.10/  0.18 | 0.990 | 1.24/  0.25 | 1.14/  0.22 | 0.075 |
| Resistance Index | 0.52/  0.05 | 0.54/  0.05 | 0.029 | 0.57/  0.06 | 0.56/  0.05 | 0.407 | 0.60/  0.07 | 0.62/  0.05 | 0.103 | 0.64/  0.07 | 0.65/  0.06 | 0.349 | 0.68/  0.06 | 0.66/  0.06 | 0.083 |
| Cerebrovascular resistance index | 1.74/  0.39 | 1.42/  0.32 | <0.001 | 2.18/  0.72 | 1.69/  0.44 | <0.001 | 2.27/  0.59 | 2.08/  0.58 | 0.026 | 2.41/  0.66 | 2.37/  0.63 | 0.679 | 2.56/  0.78 | 2.85/  0.93 | 0.169 |

Supplementary Table 1. Demographic, clinical and physiological variables stratified by sex and age for the group of patients with End-tidal CO_2_ measures. Values are given as Mean/SD unless otherwise specified.

|  | <55  years of age | | | 55-64  years of age | | | 65-74  years of age | | | 75-84  years of age | | | ≥85  years of age | | |
| --- | --- | --- | --- | --- | --- | --- | --- | --- | --- | --- | --- | --- | --- | --- | --- |
|  | Males | Females | *p* | Males | Females | *p* | Males | Females | *p* | Males | Females | *p* | Males | Females | *p* |
| N | 51 | 39 |  | 27 | 29 |  | 37 | 23 |  | 22 | 30 |  | 6 | 21 |  |
| Age | 43.08/  8.36 | 43.51/  8.96 | 0.813 | 59.44/  3.07 | 59.31/  3.00 | 0.869 | 69.19/  2.83 | 69.26/  2.85 | 0.924 | 80.86/  2.98 | 78.73/  3.15 | 0.017 | 88.17/  2.64 | 89.19/  4.11 | 0.572 |
| Haemoglobin (g/dL) | 15.44/  1.06 | 13.46/  0.94 | <0.001 | 14.63/  1.46 | 13.40/  1.08 | <0.001 | 14.50/  1.71 | 13.62/  1.28 | 0.037 | 13.93/  1.69 | 13.02/  1.41 | 0.04 | 12.68/  1.74 | 13.07/  1.33 | 0.565 |
| Haematocrit | 0.45/  0.03 | 0.40/  0.02 | <0.001 | 0.43/  0.04 | 0.40/  0.04 | 0.004 | 0.43/  0.04 | 0.41/  0.04 | 0.084 | 0.42/  0.05 | 0.40/  0.04 | 0.132 | 0.38/  0.05 | 0.39/  0.04 | 0.564 |
| Systolic blood pressure (mmHg) | 129.00/  11.23 | 127.24/  14.46 | 0.518 | 124.43/  1.00 | 129.38/  15.07 | 0.156 | 139.55/  17.36 | 141.50/  20.02 | 0.692 | 138.50/  16.41 | 146.80/  23.47 | 0.161 | 145.67/  19.51 | 157.26/  19.73 | 0.215 |
| Diastolic blood pressure (mmHg) | 77.92/  7.37 | 76.38/  8.07 | 0.349 | 78.65/  6.84 | 76.10/  8.38 | 0.221 | 79.70/  9.45 | 75.43/  8.41 | 0.082 | 77.70/  11.47 | 76.08/  10.80 | 0.605 | 78.58/  14.86 | 83.24/  11.12 | 0.409 |
| Mean blood pressure (mmHg) | 94.76/  7.79 | 93.10/  9.21 | 0.357 | 93.70/  6.91 | 93.57/  9.41 | 0.954 | 99.39/  11.16 | 97.15/  10.80 | 0.447 | 97.77/  12.30 | 99.32/  13.72 | 0.676 | 100.78/  16.15 | 107.68/  11.03 | 0.234 |
| Heart rate (bpm) | 68.24/  12.92 | 72.09/  14.22 | 0.183 | 66.58/  12.28 | 69.50/  9.64 | 0.328 | 66.70/  14.58 | 69.57/  9.45 | 0.406 | 65.00/  11.38 | 74.03/  8.73 | 0.002 | 65.42/  9.65 | 78.29/  10.19 | 0.011 |
| Peak systolic velocity (cm/s) | 90.66/  13.69 | 107.12/  15.72 | <0.001 | 83.61/  16.70 | 92.76/  17.24 | 0.049 | 83.22/  16.40 | 77.41/  13.53 | 0.160 | 72.76/  20.50 | 78.76/  20.50 | 0.259 | 92.33/  13.75 | 63.24/  17.07 | <0.001 |
| End-diastolic velocity (cm/s) | 42.70/  7.69 | 49.08/  8.90 | <0.001 | 30.04/  6.49 | 40.80/  8.44 | 0.177 | 34.46/  7.97 | 30.70/  7.63 | 0.076 | 27.41/  6.76 | 27.99/  7.67 | 0.777 | 31.54/  7.76 | 22.42/  5.42 | 0.003 |
| Mean flow velocity (cm/s) | 60.24/  9.48 | 72.57/  11.43 | <0.001 | 55.21/  10.35 | 61.98/  11.73 | 0.026 | 52.76/  10.97 | 48.68/  10.17 | 0.156 | 43.18/  11.53 | 47.72/  11.16 | 0.160 | 53.33/  8.35 | 38.38/  10.03 | 0.003 |
| Pulsatility Index | 0.80/  0.11 | 0.81/  0.13 | 0.852 | 0.82/  0.10 | 0.84/  0.10 | 0.492 | 0.94/  0.15 | 0.98/  0.18 | 0.348 | 1.05/  0.21 | 1.09/  0.22 | 0.515 | 1.16/  0.28 | 1.05/  0.18 | 0.261 |
| Resistance Index | 0.53/  0.04 | 0.53/  0.04 | 0.259 | 0.54/  0.04 | 0.56/  0.04 | 0.101 | 0.59/  0.05 | 0.60/  0.06 | 0.192 | 0.60/  0.11 | 0.65/  0.07 | 0.086 | 0.66/  0.08 | 0.64/  0.07 | 0.522 |
| Cerebrovascular resistance index | 1.61/  0.29 | 1.61/  0.29 | <0.001 | 1.75/  0.36 | 1.58/  0.43 | 0.110 | 1.98/  0.54 | 2.09/  0.54 | 0.449 | 2.45/  0.83 | 2.23/  0.75 | 0.327 | 1.92/  0.40 | 3.03/  0.98 | 0.013 |

Supplementary Table 2. Demographic, clinical and physiological variables stratified by sex and age excluding patients on antihypertensive medications at the time of assessment. Values are given as Mean/SD unless otherwise specified.

|  | <55  years of age | | | 55-64  years of age | | | 65-74  years of age | | | ≥75  years of age | | | ≥85  years of age | | |
| --- | --- | --- | --- | --- | --- | --- | --- | --- | --- | --- | --- | --- | --- | --- | --- |
|  | Males | Females | *p* | Males | Females | *p* | Males | Females | *p* | Males | Females | *p* | Males | Females | *p* |
| N | 111 | 63 |  | 83 | 71 |  | 152 | 110 |  | 118 | 134 |  | 45 | 55 |  |
| Age | 45.86/  7.43 | 44.44/  7.81 | 0.236 | 59.53/  2.74 | 59.69/  3.01 | 0.730 | 69.83/  2.76 | 69.27/  2.97 | 0.120 | 79.88/  2.79 | 78.97/  2.98 | 0.013 | 88.78/  2.46 | 88.25/  3.36 | 0.386 |
| Haemoglobin (g/dL) | 15.33/  1.03 | 13.36/  0.94 | <0.001 | 14.69/  1.45 | 13.54/  1.31 | <0.001 | 14.68/  1.51 | 13.63/  1.28 | <0.001 | 13.92/  1.49 | 13.20/  1.25 | <0.001 | 13.30/  1.86 | 13.18/  1.25 | 0.702 |
| Haematocrit | 0.45/  0.03 | 0.40/  0.03 | <0.001 | 0.44/  0.04 | 0.41/  0.04 | <0.001 | 0.44/  0.04 | 0.41/  0.04 | <0.001 | 0.42/  0.04 | 0.40/  0.04 | <0.001 | 0.40/  0.05 | 0.40/  0.04 | 0.492 |
| Systolic blood pressure (mmHg) | 135.95/  17.87 | 133.29/  16.72 | 0.335 | 138.80/  19.56 | 136.68/  19.49 | 0.504 | 144 82/  19.84 | 148.71/  20.27 | 0.122 | 146.35/  17.57 | 154.66/  23.95 | 0.002 | 151.87/  21.17 | 158.75/  22.07 | 0.118 |
| Diastolic blood pressure (mmHg) | 82.90/  11.10 | 80.41/  9.54 | 0.138 | 83.43/  11.09 | 79.51/  10.08 | 0.024 | 81.65/  11.89 | 79.65/  10.19 | 0.157 | 79.28/  11.66 | 80.03/  12.08 | 0.617 | 78.48/  12.94 | 81.20/  12.45 | 0.288 |
| Mean blood pressure (mmHg) | 100.38/  12.61 | 97.79/  10.74 | 0.172 | 101.63/  12.82 | 98.32/  11.95 | 0.103 | 102.47/  13.32 | 102.23/  12.42 | 0.881 | 101.60/  12.16 | 104.66/  14.39 | 0.073 | 102.70/  13.32 | 106.91/  13.52 | 0.122 |
| Heart rate (bpm) | 69.47/  12.36 | 72.75/  14.82 | 0.119 | 69.58/  14.20 | 72.82/  12.05 | 0.135 | 67.84/  13.31 | 71.19/  12.06 | 0.038 | 66.56/  11.38 | 75.14/  11.43 | <0.001 | 67.11/  12.79 | 72.64/  11.74 | 0.027 |
| Peak systolic velocity (cm/s) | 87.05/  15.21 | 102.22/  18.75 | <0.001 | 78.12/  15.82 | 89.35/  16.23 | <0.001 | 76.49/  15.06 | 81.98/  15.51 | 0.004 | 73.68/  16.95 | 76.63/  15.98 | 0.158 | 78.38/  18.19 | 70.96/  19.26 | 0.052 |
| End-diastolic velocity (cm/s) | 41.22/  8.00 | 47 36/  9.51 | <0.001 | 33.92/  7.58 | 38.98/  8.34 | <0.001 | 30.21/  7.39 | 31.50/  7.43 | 0.167 | 26.37/  6.77 | 26.60/  6.30 | 0.783 | 24.41/  7.56 | 23.27/  6.33 | 0.412 |
| Mean flow velocity (cm/s) | 58.24/  10.37 | 69.67/  13.13 | <0.001 | 50.02/  10.80 | 59.21/  11.37 | <0.001 | 47.25/  9.93 | 51.42/  10.56 | <0.001 | 43.38/  10.01 | 45.65/  9.70 | 0.069 | 43.68/  10.95 | 41.20/  10.64 | 0.255 |
| Pulsatility Index | 0.79/  0.14 | 0.79/  0.13 | 0.983 | 0.90/  0.17 | 0.86/  0.12 | 0.134 | 0.99/  0.21 | 0.99/  0.16 | 0.934 | 1.10/  0.23 | 1.10/  0.18 | 0.899 | 1.26/  0.26 | 1.16/  0.23 | 0.043 |
| Resistance Index | 0.53/  0.05 | 0.54/  0.05 | 0.249 | 0.57/  0.06 | 0.56/  0.05 | 0.708 | 0.60/  0.07 | 0.62/  0.06 | 0.124 | 0.64/  0.08 | 0.65/  0.06 | 0.132 | 0.69/  0.07 | 0.67/  0.07 | 0.120 |
| Cerebrovascular resistance index | 1.78/  0.39 | 1.45/  0.32 | <0.001 | 2.15/  0.68 | 1.73/  0.45 | <0.001 | 2.27/  0.59 | 2.09/  0.59 | 0.014 | 2.48/  0.71 | 2.41/  0.70 | 0.469 | 2.51/  0.75 | 2.79/  0.87 | 0.094 |

Supplementary Table 3. Demographic, clinical and physiological variables stratified by sex and age excluding female patients on hormone replacement therapy for menopause at the time of assessment. Values are given as Mean/SD unless otherwise specified.

|  | <55  years of age | | | 55-64  years of age | | | 65-74  years of age | | | 75-84  years of age | | | ≥85  years of age | | |
| --- | --- | --- | --- | --- | --- | --- | --- | --- | --- | --- | --- | --- | --- | --- | --- |
|  | Males | Females | *p* | Males | Females | *p* | Males | Females | *p* | Males | Females | *p* | Males | Females | *p* |
| N | 76 | 50 |  | 62 | 60 |  | 124 | 104 |  | 117 | 131 |  | 43 | 53 |  |
| Age | 46.53/  7.20 | 45.24/  7.76 | 0.343 | 59.52/  2.75 | 60.00/  2.96 | 0.351 | 70.06/  2.66 | 69.36/  2.97 | 0.061 | 79.89/  2.80 | 79.02/  2.92 | 0.018 | 88.79/  2.51 | 88.30/  3.42 | 0.436 |
| Haemoglobin (g/dL) | 15.27/  0.94 | 13.30/  0.97 | <0.001 | 14.63/  1.45 | 13.50/  1.29 | <0.001 | 14.76/  1.47 | 13.53/  1.25 | <0.001 | 13.92/  1.49 | 13.20/  1.24 | <0.001 | 13.26/  1.88 | 13.20/  1.26 | 0.872 |
| Haematocrit | 0.45/  0.03 | 0.40/  0.03 | <0.001 | 0.43/  0.04 | 0.41/  0.04 | <0.001 | 0.44/  0.04 | 0.41/  0.04 | <0.001 | 0.42/  0.04 | 0.40/  0.04 | <0.001 | 0.40/  0.05 | 0.40/  0.04 | 0.683 |
| Systolic blood pressure (mmHg) | 136.55/  17.79 | 133.71/  16.34 | 0.367 | 137.50/  17.76 | 136.66/  19.63 | 0.803 | 144.77/  20.36 | 148.96/  20.93 | 0.128 | 146.28/  17.63 | 154.22/  23.86 | 0.004 | 152.16/  21.08 | 158.06/  21.23 | 0.178 |
| Diastolic blood pressure (mmHg) | 83.66/  11.29 | 79.68/  9.04 | 0.039 | 83.40/  12.18 | 79.03/  10.35 | 0.036 | 81.45/  11.23 | 79.51/  10.74 | 0.176 | 79.22/  11.70 | 80.13/  12.22 | 0.552 | 79.10/  12.89 | 81.16/  12.6 | 0.435 |
| Mean blood pressure (mmHg) | 101.08/  12.76 | 97.40/  10.21 | 0.090 | 101.17/  13.18 | 98.00/  12.12 | 0.173 | 102.36/  13.14 | 102.42/  12.83 | 0.968 | 101.54/  12.19 | 104.58/  14.43 | 0.078 | 103.21/  13.28 | 106.65/  13.60 | 0.217 |
| Heart rate (bpm) | 69.41/  12.11 | 71.88/  13.62 | 0.288 | 68.38/  14.44 | 72.35/  12.02 | 0.104 | 67.28/  13.52 | 70.83/  11.84 | 0.038 | 66.65/  11.39 | 75.23/  11.61 | <0.001 | 67.58/  12.88 | 72.14/  10.91 | 0.063 |
| Peak systolic velocity (cm/s) | 86.59/  14.35 | 103.10/  19.46 | <0.001 | 78.30/  14.93 | 90.13/  15.29 | <0.001 | 76.98/  15.50 | 82.80/  15.42 | 0.005 | 73.73/  17.02 | 76.15/  15.93 | 0.247 | 77.93/  18.49 | 70.00/  19.96 | 0.042 |
| End-diastolic velocity (cm/s) | 41.24/  8.09 | 47.37/  9.27 | <0.001 | 34.25/  7.22 | 39.35/  8.02 | <0.001 | 30.30/  7.44 | 31.60/  7.45 | 0.192 | 26.40/  6.80 | 26.62/  6.34 | 0.793 | 24.51/  7.72 | 22.92/  6.17 | 0.266 |
| Mean flow velocity (cm/s) | 58.23/  10.19 | 70.06/  13.33 | <0.001 | 50.15/  10.35 | 59.78/  10.80 | <0.001 | 47.47/  10.12 | 51.74/  10.53 | 0.002 | 43.41/  10.05 | 45.53/  9.76 | 0.094 | 43.62/  11.17 | 40.59/  10.34 | 0.172 |
| Pulsatility Index | 0.79/  0.13 | 0.80/  0.13 | 0.586 | 0.89/  0.18 | 0.86/  0.13 | 0.245 | 1.00/  0.22 | 1.00/  0.17 | 0.902 | 1.10/  0.24 | 1.10/  0.18 | 0.891 | 1.25/  0.26 | 1.16/  0.23 | 0.081 |
| Resistance Index | 0.52  /0.05 | 0.54/  0.06 | 0.143 | 0.56/  0.06 | 0.56/  0.05 | 0.916 | 0.60/  0.07 | 0.61/  0.06 | 0.098 | 0.64/  0.08 | 0.65/  0.06 | 0.208 | 0.68/  0.07 | 0.67/  0.07 | 0.194 |
| Cerebrovascular resistance index | 1.79  /0.40 | 1.44/  0.32 | <0.001 | 2.13/  0.68 | 1.70/  0.41 | <0.001 | 2.26/  0.56 | 2.07/  0.54 | 0.012 | 2.48/  0.71 | 2.42/  0.71 | 0.548 | 2.53/  0.76 | 2.82/  0.87 | 0.093 |
| **Subgroup with End-tidal CO2** | | | | | | | | | | | | |  |  |  |
| N | 56 | 40 |  | 43 | 41 |  | 87 | 77 |  | 81 | 93 |  | 32 | 36 |  |
| N | 45.61/  7.80 | 44.67/  7.94 | 0.568 | 59.44/  2.80 | 60.27/  2.98 | 0.194 | 70.05/  2.65 | 69.56/  2.83 | 0.256 | 80.01/  2.77 | 79.12/  2.97 | 0.043 | 88.81/  2.53 | 87.72/  2.67 | 0.090 |
| Age | 15.24/  0.96 | 13.20/  0.82 | <0.001 | 14.62/  1.54 | 13.57/  1.36 | 0.001 | 14.75/  1.49 | 13.64/  1.14 | <0.001 | 13.99/  1.46 | 13.23/  1.27 | <0.001 | 13.36/  1.73 | 13.33/  1.21 | 0.917 |
| Haemoglobin (g/dL) | 0.45/  0.03 | 0.39/  0.03 | <0.001 | 0.43/  0.04 | 0.41/  0.04 | 0.005 | 0.44/  0.04 | 0.41/  0.03 | <0.001 | 0.42/  0.04 | 0.40/  0.04 | 0.002 | 0.40/  0.05 | 0.40/  0.04 | 0.847 |
| Haematocrit | 134.65/  16.84 | 131.90/  16.81 | 0.432 | 138.26/  18.80 | 135.10/  17.45 | 0.428 | 143.74/  19.66 | 147.62/  20.20 | 0.215 | 144.04/  16.56 | 153.73/  21.25 | 0.001 | 148.33/  18.71 | 155.92/  20.82 | 0.120 |
| Systolic blood pressure (mmHg) | 82.68/  11.46 | 78.71/  9.42 | 0.075 | 83.99/  13.81 | 78.79/  10.91 | 0.060 | 81.23/  10.88 | 78.97/  10.54 | 0.179 | 78.59/  11.85 | 80.11/  12.40 | 0.414 | 79.52/  12.83 | 81.06/  11.93 | 0.610 |
| Diastolic blood pressure (mmHg) | 99.71/  12.53 | 96.11/  10.46 | 0.141 | 101.73/  14.84 | 97.24/  11.69 | 0.130 | 101.75/  12.67 | 101.56/  12.48 | 0.921 | 100.36/  11.67 | 104.34/  13.72 | 0.044 | 102.16/  12.66 | 105.65/  13.33 | 0.273 |
| Mean blood pressure (mmHg) | 69.58/  11.37 | 70.08/  12.92 | 0.843 | 69.48/  16.36 | 71.50/  10.42 | 0.504 | 66.09/  12.79 | 69.90/  11.46 | 0.048 | 66.08/  11.82 | 74.50/  11.64 | <0.001 | 66.70/  12.41 | 69.67/  9.88 | 0.278 |
| End-tidal CO2 (KPa) | 5.47/  0.45 | 5.22/  0.63 | 0.026 | 5.09/  0.54 | 5.31/  0.61 | 0.082 | 5.03/  0.59 | 5.20/  0.60 | 0.069 | 4.92/  0.63 | 5.05/  0.55 | 0.149 | 4.68/  0.70 | 4.88/  0.69 | 0.258 |
| Peak systolic velocity (cm/s) | 87.61/  14.22 | 103.94/  20.28 | <0.001 | 78.44/  15.95 | 90.43/  16.22 | <0.001 | 76.19/  15.07 | 82.39/  14.56 | 0.008 | 74.64/  17.26 | 76.53/  15.07 | 0.443 | 75.86/  18.61 | 68.72/  18.62 | 0.119 |
| End-diastolic velocity (cm/s) | 42.30/  8.20 | 47.28/  9.69 | 0.008 | 34.57/  7.30 | 40.04/  8.73 | 0.002 | 30.20/  6.93 | 31.61/  6.85 | 0.193 | 26.76/  6.92 | 26.90/  6.00 | 0.883 | 24.16/  7.43 | 23.22/  6.72 | 0.582 |
| Mean flow velocity (cm/s) | 59.13/  10.36 | 70.27/  13.86 | <0.001 | 50.37/  10.75 | 60.46/  11.53 | <0.001 | 46.87/  9.49 | 51.32/  9.68 | 0.003 | 43.93/  10.11 | 45.73/  9.27 | 0.221 | 42.51/  10.89 | 40.08/  11.06 | 0.367 |
| Pulsatility Index | 0.78/  0.13 | 0.81/  0.14 | 0.261 | 0.88/  0.18 | 0.84/  0.13 | 0.278 | 0.99/  0.23 | 1.00/  0.16 | 0.906 | 1.10/  0.25 | 1.09/  0.18 | 0.847 | 1.24/  0.25 | 1.14/  0.22 | 0.089 |
| Resistance Index | 0.52/  0.05 | 0.54/  0.06 | 0.031 | 0.56/  0.06 | 0.56/  0.05 | 0.844 | 0.60/  0.07 | 0.62/  0.05 | 0.117 | 0.64/  0.07 | 0.65/  0.05 | 0.473 | 0.68/  0.06 | 0.66/  0.06 | 0.108 |
| Cerebrovascular resistance index | 1.74/  0.40 | 1.42/  0.32 | <0.001 | 2.15/  0.73 | 1.67/  0.43 | <0.001 | 2.26/  0.55 | 2.05/  0.51 | 0.013 | 2.41/  0.66 | 2.39/  0.63 | 0.885 | 2.58/  0.79 | 2.85/  0.93 | 0.200 |

Supplementary Table 4. Demographic, clinical and physiological variables stratified by sex and age excluding current smokers in the whole cohort and in the subgroup with End-tidal CO_2_ measures

|  | **R^2^** | **p** |
| --- | --- | --- |
| Peak systolic velocity/Haemoglobin | | |
| Age<55 | 0.207 | <0.001 |
| Age 55-64 | 0.127 | <0.001 |
| Age ≥65 | 0.039 | <0.001 |
| End-diastolic velocity/ Haemoglobin | | |
| Age<55 | 0.101 | <0.001 |
| Age 55-64 | 0.052 | 0.002 |
| Age ≥65 | 0.003 | 0.107 |
| Mean flow velocity/ Haemoglobin | | |
| Age<55 | 0.199 | <0.001 |
| Age 55-64 | 0.100 | <0.001 |
| Age ≥65 | 0.023 | <0.001 |
| Pulsatility Index/ Haemoglobin | | |
| Age<55 | 0.007 | 0.131 |
| Age 55-64 | 0.005 | 0.191 |
| Age ≥65 | 0.004 | 0.054 |
| Resistance Index/ Haemoglobin | | |
| Age<55 | 0.034 | 0.008 |
| Age 55-64 | 0.015 | 0.069 |
| Age ≥65 | 0.017 | <0.001 |
| Cerebrovascular resistance index/ Haemoglobin | | |
| Age<55 | 0.190 | <0.001 |
| Age 55-64 | 0.089 | <0.001 |
| Age ≥65 | 0.045 | <0.001 |

Supplementary Table 5. Univariate regression analysis performed with each transcranial Doppler parameter as dependent variable, and haemoglobin as independent variable, stratified by age.

| Independent Variables | **Model A** | | **Model B** | | **Model C** | | **Model D** | |
| --- | --- | --- | --- | --- | --- | --- | --- | --- |
|  | B (95% CI) | *p* | B (95% CI) | *p* | B (95% CI) | *p* | B (95% CI) | *p* |
| Female vs male | **3.71 (1.56,5.87)** | <0.001 | **0.88 (-1.37,3.13)** | 0.104 | **3.37 (0.83,5.91)** | 0.009 | **0.12 (2.51,2.75)** | 0.927 |
| Age | -0.61 (-0.69,-0.53) | <0.001 | -0.67 (-0.75,-0.59) | <0.001 | -0.59 (-0.68, -0.49) | <0.001 | -0.64 (0.73,0.55) | <0.001 |
| Mean systolic blood pressure (mmHg) | 0.22 (0.16,0.29) | <0.001 | 0.22 (0.16,0.28) | <0.001 | 0.24 (0.16, 0.32) | <0.001 | 0.24 (0.16,0.32) | <0.001 |
| Mean diastolic blood pressure (mmHg) | -0.62 (-0.74,-0.50) | <0.001 | -0.54 (-0.65,-0.42) | <0.001 | -0.67 (-0.81,-0.54) | <0.001 | -0.59 (-0.72,-0.45) | <0.001 |
| Mean heart rate (bpm) | 0.08 (-0.01,0.17) | 0.067 | 0.07 (-0.01,0.16) | 0.080 | 0.14 (-0.04,0.25) | 0.006 | 0.14 (-0.04,0.24) | 0.006 |
| End-tidal CO_2_ (KPa) |  | | | | 0.23 (0.23,4.24) | 0.029 | 2.28 (0.34,4.22) | 0.021 |
| Hypertension | -1.98 (-4.24,0.28) | 0.086 | -2.16 (-4.37,0.04) | 0.055 | -3.18 (-5.83,0.53) | 0.019 | -3.27 (-5.83,-0.71) | 0.013 |
| Diabetes | -1.61 (-4.87,1.65) | 0.333 | -2.80 (-5.99,0.40) | 0.086 | -0.62 (-4.43,3.18) | 0.747 | -2.08 (-5.78,1.62) | 0.270 |
| Hyperlipidaemia | 1.05 (-1.21,3.31) | 0.360 | 0.77 (-1.44,2.97) | 0.495 | 1.05 (-1.59,3.69) | 0.437 | 0.81 (-1.75,3.36) | 0.536 |
| Smoking history | -2.27 (-4.34,-0.19) | 0.032 | -1.88 (-3.91,0.14) | 0.068 | -1.35 (-3.77,1.07) | 0.273 | -0.98 (-3.32,1.37) | 0.412 |
| Haemoglobin |  | | -2.69 (-3.45,-1.93) | <0.001 |  | | -3.04 (-3.91,-2.16) | <0.001 |
| Constant | 131.23 (120.76,141.70) | <0.001 | 170.96 (155.83,186.10) | <0.001 | 115.74 (98.61,132.88) | <0.001 | 160.72 (139.70,181.74) | <0.001 |
| N | 958 |  | 958 |  | 690 |  | 690 |  |
| Adjusted R^2^ | 0.254 |  | 0.298 |  | 0.291 |  | 0.336 |  |

Supplementary Table 6A. Multivariate regression analysis performed on the whole cohort and in the group with End-tidal CO_2_ measures, using peak systolic velocity as dependent variable, and sex (female vs male), age, systolic and diastolic blood pressure, heart rate, history of hypertension, diabetes, hyperlipidaemia and smoking habit as independent variables (Model A). Model B included hemoglobin as one of the independent variables, to explore its effect on the correlation between sex and peak systolic velocity. Model C and D included End-tidal CO_2_ (C) and End-tidal CO_2_ and haemoglobin (D) as independent variables in the group of patients with End-tidal CO_2_ measures.

| Independent Variables | **Model A** | | **Model B** | | **Model C** | | **Model D** | |
| --- | --- | --- | --- | --- | --- | --- | --- | --- |
|  | B (95% CI) | *p* | B (95% CI) | *p* | B (95% CI) | *p* | B (95% CI) | *p* |
| Female vs male | **15.89 (10.97,20.80)** | <0.001 | **6.92 (0.45,13.40)** | 0.036 | **16.33 (10.43,22.22)** | <0.001 | **5.92 (-1.81,13.65)** | 0.132 |
| Age | -0.50 (-0.81,-0.19) | <0.001 | -0.53 (-0.83,-0.23) | <0.001 | -0.51 (-0.86,-0.16) | 0.004 | -0.54 (-0.87,-0.21) | 0.001 |
| Mean systolic blood pressure (mmHg) | 0.31 (0.11,0.50) | 0.002 | 0.28 (0.10,0.47) | 0.003 | 0.37 (0.14,0.60) | 0.002 | 0.32 (0.11,0.54) | 0.004 |
| Mean diastolic blood pressure (mmHg) | -0.44 (-0.78,-0.09) | 0.014 | -0.35 (-0.68,-0.01) | 0.042 | -0.52 (-0.94,-0.11) | 0.014 | -0.40 (-0.80,-0.004) | 0.048 |
| Mean heart rate (bpm) | 0.17 (-0.02,0,37) | 0.078 | 0.21 (0.02,0.39) | 0.030 | 0.18 (-0.5,0.41) | 0.130 | 0.23 (0.01,0.45) | 0.040 |
| End-tidal CO_2_ (KPa) |  | | | | 1.56 (-3.52,6.64) | 0.544 | 0.92 (-3.91,5.74) | 0.707 |
| Hypertension | -5.53 (-11.41,0.36) | 0.066 | -6.70 (-12.38,-1.03) | 0.021 | -6.65 (-13.85,0.55) | 0.070 | -7.72 (-14.57,-0.87) | 0.027 |
| Diabetes | 6.11 (-3.26,15.48) | 0.199 | 2.69 (-6.45,11.83) | 0.562 | 6.81 (-3.31,16.93) | 0.186 | 3.41(-6.35,13.17) | 0.490 |
| Hyperlipidaemia | -0.21 (-6.75,6.33) | 0.949 | 1.76 (-4.58,8.11) | 0.584 | -2.51 (-10.74,5.72) | 0.548 | -0.42 (-8.30,7.46) | 0.916 |
| Smoking history | -1.57 (-6.27,3.12) | 0.509 | -1.22 (-5.73,3.29) | 0.594 | -0.69 (-6.32,4.95) | 0.809 | -0.40 (-5.75,4.95) | 0.882 |
| Haemoglobin |  | | -4.63 (-6.93,-2.34) | <0.001 |  | | -5.18 (-7.84,-2.53) | <0.001 |
| Constant | 77.43 (51.49,103.38) | <0.001 | 152.92 (107.98,197.86) | <0.001 | 68.35 (25.62,11.08) | 0.002 | 155.73 (95.32,216.14) | <0.001 |
| N | 181 |  | 181 |  | 137 |  | 137 |  |
| Adjusted R^2^ | 0.288 |  | 0.346 |  | 0.300 |  | 0.371 |  |

Supplementary Table 6B. Multivariate regression analysis performed in all patients aged <55 years, and in the group with End-tidal CO_2_ measures, using peak systolic velocity as dependent variable, and sex (female vs male), age, systolic and diastolic blood pressure, heart rate, history of hypertension, diabetes, hyperlipidaemia and smoking habit as independent variables (Model A). Model B included hemoglobin as one of the independent variables, to explore its effect on the correlation between sex and peak systolic velocity. Model C and D included End-tidal CO_2_ (C) and End-tidal CO_2_ and haemoglobin (D) as independent variables in the group of patients with End-tidal CO_2_ measures.

| Independent Variables | **Model A** | | **Model B** | | **Model C** | | **Model D** | |
| --- | --- | --- | --- | --- | --- | --- | --- | --- |
|  | B (95% CI) | *p* | B (95% CI) | *p* | B (95% CI) | *p* | B (95% CI) | *p* |
| Female vs male | **7.96 (2.63,13.29)** | 0.004 | **5.10 (-0.54,10.74)** | 0.076 | **8.18 (1.63,14.74)** | 0.015 | **5.01 (-1.90,11.92)** | 0.153 |
| Age | 0.18 (-0.71,1.07) | 0.684 | 0.30 (-0.57, 1.17) | 0.499 | 0.10 (-0.96,1.17) | 0.846 | 0.25 (-0.80,1.29) | 0.641 |
| Mean systolic blood pressure (mmHg) | 0.002 (-0.18,0.18) | 0.983 | -0.02 (-0.19,0.16) | 0.854 | -0.4 (-0.26,0.18) | 0.713 | -0.07 (-0.29,0.14) | 0.505 |
| Mean diastolic blood pressure (mmHg) | -0.48 (-0.83,-0.13) | 0.008 | -0.38 (-0.73,-0.03) | 0.033 | -0.50 (-0.90,-0.11) | 0.013 | -0.38 (-0.78,0.02) | 0.061 |
| Mean herat rate (bpm) | 0.13 (-0.09,0.34) | 0.253 | 0.10 (-0.11,0.31) | 0.344 | 0.27 (-0.005,0.54) | 0.054 | 0.23 (-0.04,0.50) | 0.097 |
| End-tidal CO_2_ (KPa) |  | | | | 3.59 (-2.13,9.31) | 0.216 | 4.67 (-0.99,10.33) | 0.104 |
| Hypertension | 0.64 (-5.05,6.34) | 0.824 | 0.06 (-5.54,5.65) | 0.984) | 0.29 (-6.58,7.16) | 0.933 | 0.14 (-6.57,6.85) | 0.967 |
| Diabetes | -0.21 (-7.87,7.44) | 0.956 | -2.13 (-9.77,5.50) | 0.582 | 1.64 (-7.88,11.17) | 0.733 | -0.01 (-9.41,9.39) | 0.998 |
| Hyperlipidaemia | 0.85 (-5.16,6.86) | 0.781 | -0.15 (-6.08,5.78) | 0.960 | 0.11 (-7.14,7.35) | 0.977 | -1.16 (-8.31,5.99) | 0.749 |
| Smoking history | -3.74 (-9.04,1.56) | 0.165 | -2.78 (-8.02,2.46) | 0.297 | -1.76 (-8.34,4.34) | 0.598 | -0.71 (-7.20,5.78) | 0.829 |
| Haemoglobin |  | | -2.62 (-4.57,-0.66) | 0.009 |  | | -2.71 (-4.94,-0.48) | 0.018 |
| Constant | 91.83 (34.56,149.11) | 0.002 | 122.91 (62.19,183.63) | <0.001 | 75.56 (-3.62,154.74) |  | 102.02 (21.71,182.33) | 0.013 |
| N | 155 |  | 155 |  | 110 |  | 110 |  |
| Adjusted R^2^ | 0.134 |  | 0.169 |  | 0.193 |  | 0.231 |  |

Supplementary Table 6C. Multivariate regression analysis performed in all patients aged 55-64 years, and in the group with EtCO_2_ measures, using peak systolic velocity as dependent variable, and sex (female vs male), age, systolic and diastolic blood pressure, heart rate, history of hypertension, diabetes, hyperlipidaemia and smoking habit as independent variables (Model A). Model B included hemoglobin as one of the independent variables, to explore its effect on the correlation between sex and peak systolic velocity. Model C and D included End-tidal CO_2_ (C) and End-tidal CO_2_ and haemoglobin (D) as independent variables in the group of patients with End-tidal CO_2_ measures.

| Independent Variables | **Model A** | | **Model B** | | **Model C** | | **Model D** | |
| --- | --- | --- | --- | --- | --- | --- | --- | --- |
|  | B (95% CI) | *p* | B (95% CI) | *p* | B (95% CI) | *p* | B (95% CI) | *p* |
| Female vs male | **-0.64 (-3.30,2.03)** | 0.638 | **-2.31 (-5.03,0.41)** | 0.096 | **-2.21 (-5.32,0.90)** | 0.163 | **-4.02 (-7.13,-0.86)\|** | 0.013 |
| Age | -0.50 (-0.66,-0.33) | <0.001 | -0.58 (-0.75,-0.41) | <0.001 | -0.41 (-0.61,-0.22) | <0.001 | -0.51 (-0.71,-0.32)\| | <0.001 |
| Mean systolic blood pressure (mmHg) | 0.26 (0.19,0.34) | <0.001 | 0.26 (0.19,0.33) | <0.001 | 0.30 (0.21,0.39) | <0.001 | 0.30 (0.21,0.39) | <0.001 |
| Mean diastolic blood pressure (mmHg) | -0.68 (-0.81,-0.54) | <0.001 | -0.61 (-0.75,-0.48) | <0.001 | -0.74 (-0.89,-0.58) | <0.001 | -0.67 (-0.83,-0.52) | <0.001 |
| Mean heart rate (bpm) | 0.041 (-0.06,0.15) | 0.439 | 0.04 (-0.07,0.14) | 0.481 | 0.12 (-0.01,0.24_ | 0.065 | 0.11 (-0.01,0.23) | 0.068 |
| End-tidal CO_2_ (KPa) |  | | | | 3.27 (0.97,5.58) | 0.006 | 3.04 (0.78,5.31) | 0.009 |
| Hypertension | -1.82 (-4.49,0.86) | 0.182 | -1.82 (-4.45,0.82) | 0.176 | -3.33 (-6.38,-0.72) | 0.033 | -3.24 (-6.23,-0.25) | 0.034 |
| Diabetes | -2.76 (-6.61,1.10) | 0.160 | -3.47 (-7.27,0.34) | 0.074 | -2.35 (-6.76,2.06) | 0.296 | -3.30 (-7.64,1.05) | 0.136 |
| Hyperlipidaemia | 1.24 (-1.33,3.80) | 0.343 | 0.96 (-1.57,3.48) | 0.458 | 1.73 (-1.19,4.65) | 0.244 | 1.50 (-1.36,4.36) | 0.303 |
| Smoking history | -3.05 (-5.59,-0.51) | 0.019 | -2.76 (-5.26,-0.25) | 0.031 | -2.27 (-5.18,0.63) | 0.125 | -2.02 (-4.87,0.83) | 0.165 |
| Haemoglobin |  | | -2.04 (-2.93,-1.15) | <0.001 |  | | -2.26 (-3.28,-1.23) | <0.001 |
| Constant | 131.61 (114.13,147.08) | <0.001 | 162.96 (141.47,184.45) | <0.001 | 104.07 (79.82,128.33) | <0.001 | 142.40 (112.93,171.87) | <0.001 |
| N | 622 |  | 622 |  | 443 |  | 443 |  |
| Adjusted R^2^ | 0.166 |  | 0.191 |  | 0.199 |  | 0.231 |  |

Supplementary Table 6D. Multivariate regression analysis performed in all patients aged ≥65 years, and in the group with EtCO_2_ measures, using peak systolic velocity as dependent variable, and sex (female vs male), age, systolic and diastolic blood pressure, heart rate, history of hypertension, diabetes, hyperlipidaemia and smoking habit as independent variables (Model A). Model B included hemoglobin as one of the independent variables, to explore its effect on the correlation between sex and peak systolic velocity. Model C and D included End-tidal CO_2_ (C) and End-tidal CO_2_ and haemoglobin (D) as independent variables in the group of patients with End-tidal CO_2_ measures.

|  | **All patients with baseline transcranial Doppler** | | | | **Patients with baseline transcranial Doppler and End-tidal CO_2_** | | | | |
| --- | --- | --- | --- | --- | --- | --- | --- | --- | --- |
|  | **Model A** | **p** | **Model B (Hb)** | **p** | **Model C (EtCO_2_)** | **p** | **Model D (EtCO_2_ and Hb)** | | **p** |
| B (95%CI) | 3.71 (1.56,5.87) | <0.001 | 0.88 (-1.37,3.13) | 0.443 | 3.37 (0.83,5.91) | 0.009 | 0.12 (-2.51,2.75) | 0.927 | |
| Adjusted R^2^ | 0.254 |  | 0.290 |  | 0.291 |  | 0.336 |  | |
| Number | 958 |  | 958 |  | 690 |  | 690 |  | |
|  | **Patients with baseline transcranial Doppler**  **and systolic blood pressure <160 mmHg** | | | | **Patients with baseline transcranial Doppler and End-tidal CO_2_**  **and systolic blood pressure <160 mmHg** | | | | |
| B (95%CI) | 5.23 (2.75,7.71) | <0.001 | 1.98 (-0.59,4.56) | 0.131 | 4.05 (1.16,6.94) | 0.006 | 0.37 (-2.59,3.33) | 0.807 | |
| Adjusted R^2^ | 0.250 |  | 0.296 |  | 0.289 |  | 0.348 |  | |
| Number | 733 |  | 733 |  | 538 |  | 538 |  | |
|  | **Analysis at one-month follow-up in patients with transcranial Doppler**  **at baseline and at one-month follow-up** | | | | **Analysis at one-month follow-up in patients with transcranial Doppler**  **at baseline and at one-monthfollow-up** | | | | |
| B (95%CI) | 3.81 (1.31,6.32) | 0.003 | 1.46 (-1.17,4.08) | 0.277 | 2.99 (0.08,5.91) | 0.044 | 0.42 (-2.63,3.46) | 0.789 | |
| Adjusted R^2^ | 0.202 |  | 0.228 |  | 0.210 |  | 0.239 |  | |
| Number | 786 |  | 786 |  | 594 |  | 594 |  | |

Supplementary Table 7: Multivariate regression analysis performed in the whole cohort, excluding severely hypertensive patients, and in an analysis repeated at one-month follow-up in patients with both baseline and one-month measures, using peak systolic velocity as dependent variable, and sex (female vs male), age, systolic and diastolic blood pressure, heart rate, history of hypertension, diabetes, hyperlipidaemia and smoking habit as independent variables (Model A). Model B included hemoglobin as one of the independent variables, to explore its effect on the correlation between sex and peak systolic velocity. Model C and D included End-tidal CO_2_ (C) and End-tidal CO_2_ and hemoglobin (D) as independent variables in the group of patients with End-tidal CO_2_ measures.

|  | **All patients with transcranial Doppler** | | | | **Patients with transcranial Doppler and End-tidal CO_2_** | | | | |
| --- | --- | --- | --- | --- | --- | --- | --- | --- | --- |
|  | **Model A** | **p** | **Model B** | **p** | **Model C** | **p** | **Model D** | | **p** |
| B (95%CI) | 1.92 (0.92,2.91) | <0.001 | 0.64 (-0.398,1.682) | 0.226 | 1.96 (0.77,3.15) | 0.001 | 0.59 (-0.65,1.83) | 0.350 | |
| Adjusted R^2^ | 0.491 |  | 0.515 |  | 0.499 |  | 0.525 |  | |
| Number | 958 |  | 958 |  | 690 |  | 690 |  | |
| **By age: <55 years** | | | | | | | | | |
| B (95%CI) | 6.60 (4.03,9.18) | <0.001 | 3.18 (-0.29,6.65) | 0.072 | 5.77 (2.70,8.84) | <0.001 | 1.16 (-2.93,5.25) | 0.575 | |
| Adjusted R^2^ | 0.221 |  | 0.252 |  | 0.170 |  | 0.228 |  | |
| Number | 181 |  | 181 |  | 137 |  | 137 |  | |
| **By Age:55-64** | | | | | | | | | |
| B (95%CI) | 4.40 (1.86,6.94) | <0.001 | 3.17 (0.47,5.887) | 0.022 | 5.21 (2.10,8.33) | 0.001 | 3.98 (0.70,7.26) | 0.018 | |
| Adjusted R^2^ | 0.192 |  | 0.218 |  | 0.241 |  | 0.266 |  | |
| Number | 155 |  | 155 |  | 110 |  | 110 |  | |
| **By Age: >=65** | | | | | | | | | |
| B (95%CI) | 0.11 (-1.04,1.27) | 0.848 | -0.71 (-1.88,0.47) | 0.240 | -0.03 (-1.40,1.34) | 0.965 | -0.83 (-2.23,0.56) | 0.242 | |
| Adjusted R^2^ | 0.221 |  | 0.252 |  | 0.209 |  | 0.240 |  | |
| Number | 622 |  | 622 |  | 443 |  | 443 |  | |

Supplementary Table 8. Multivariate regression analysis performed in the whole cohort and in the group of patients with End-tidal CO_2_ measures, using end-diastolic velocity as dependent variable, and sex (female vs male), age, systolic and diastolic blood pressure, heart rate, history of hypertension, diabetes, hyperlipidaemia and smoking habit as independent variables (Model A). Model B included hemoglobin as one of the independent variables, to explore its effect on the correlation between sex and end-diastolic velocity. Model C and D included End-tidal CO_2_ (C) and End-tidal CO_2_ and haemoglobin (D) as independent variables in the group of patients with End-tidal CO_2_ measures.

|  | **All patients with transcranial Doppler** | | | | **Patients with transcranial Doppler and End-tidal CO_2_** | | | | |
| --- | --- | --- | --- | --- | --- | --- | --- | --- | --- |
|  | **Model A** | **p** | **Model B** | **p** | **Model C** | **p** | **Model D** | | **p** |
| B (95%CI) | 4.09 (2.64,5.55) | <0.001 | 2.05 (0.54,3.56) | 0.008 | 4.05 (2.32,5.78) | <0.001 | 1.82 (0.03,3.60) | 0.046 | |
| Adjusted R^2^ | 0.350 |  | 0.386 |  | 0.368 |  | 0.409 |  | |
| Number | 958 |  | 958 |  | 690 |  | 690 |  | |
| **By age: <55 years** | | | | | | | | | |
| B (95%CI) | 12.38 (8.84,15.93) | <0.001 | 6.33 (1.64,11.03) | 0.008 | 11.71 (7.44,15.98) | <0.001 | 4.18 (-1.41,9.78) | 0.141 | |
| Adjusted R^2^ | 0.263 |  | 0.315 |  | 0.229 |  | 0.307 |  | |
| Number | 181 |  | 181 |  | 137 |  | 137 |  | |
| **By Age:55-64** | | | | | | | | | |
| B (95%CI) | 7.70 (3.99,11.41) | <0.001 | 5.79 (1.85,9.72) | 0.004 | 8.66 (4.11,13.21) | <0.001 | 6.80 (2.03,11.58) | 0.006 | |
| Adjusted R^2^ | 0.159 |  | 0.190 |  | 0.203 |  | 0.232 |  | |
| Number | 155 |  | 155 |  | 110 |  | 110 |  | |
| **By Age: >=65** | | | | | | | | | |
| B (95%CI) | 0.85 (-0.87,2.58) | 0.332 | -0.44 (-2.19,1.310 | 0.622 | 0.26 (-1.76,2.29) | 0.799 | -1.06 (-3.11,0.99) | 0.310 | |
| Adjusted R^2^ | 0.145 |  | 0.183 |  | 0.146 |  | 0.206 |  | |
| Number | 622 |  | 622 |  | 443 |  | 443 |  | |

Supplementary Table 9. Multivariate regression analysis performed in the whole cohort and in the group of patients with End-tidal CO_2_ measures, using mean flow velocity as dependent variable, and sex (female vs male), age, systolic and diastolic blood pressure, heart rate, history of hypertension, diabetes, hyperlipidaemia and smoking habit as independent variables (Model A). Model B included hemoglobin as one of the independent variables, to explore its effect on the correlation between sex and mean flow velocity. Model C and D included End-tidal CO_2_ (C) and End-tidal CO_2_ and haemoglobin (D) as independent variables in the group of patients with End-tidal CO_2_ measures.


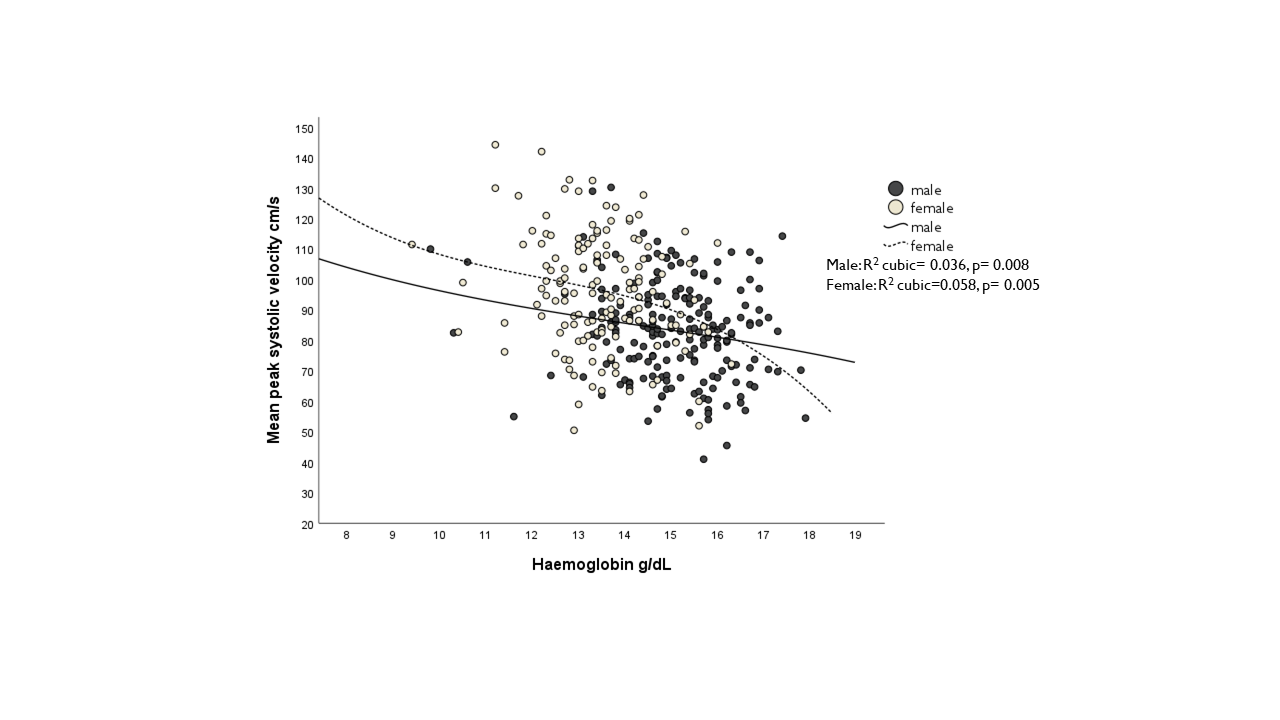


Supplementary Figure 1A: Correlation between peak systolic velocity and haemoglobin in men and women aged < 65 years.


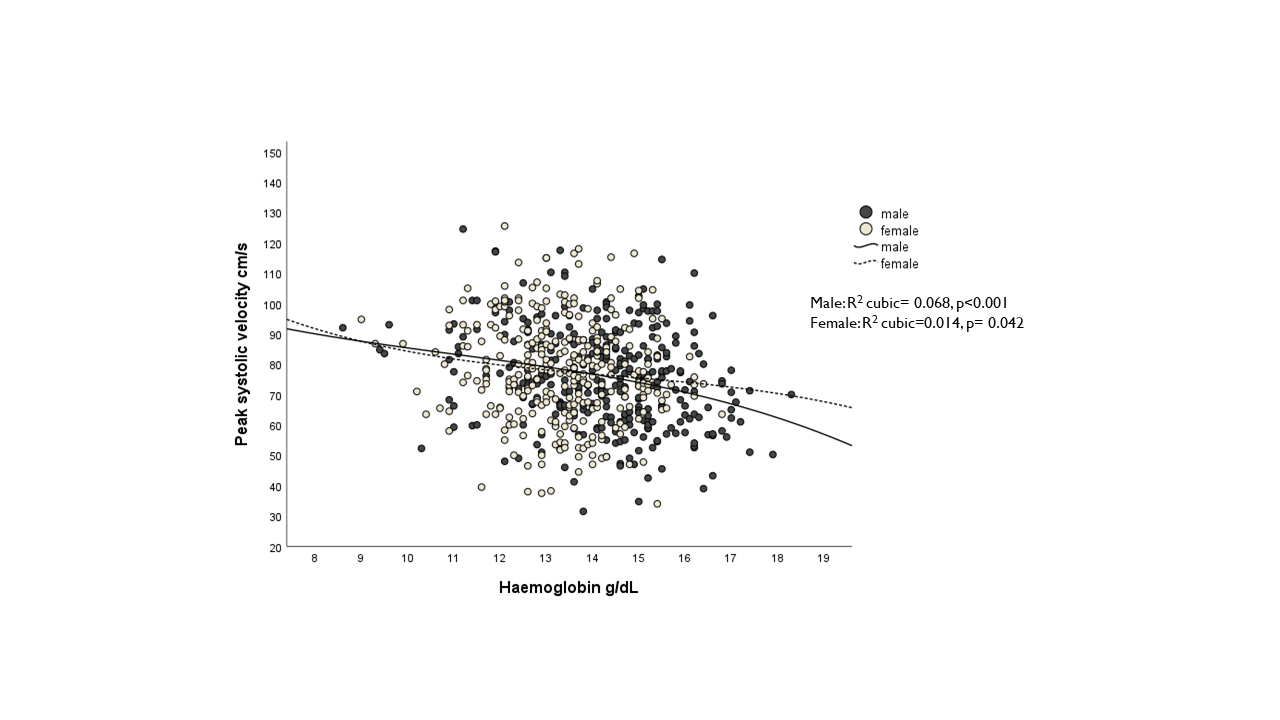


Supplementary Figure 1B: Correlation between peak systolic velocity and haemoglobin in men and women aged ≥65 years.


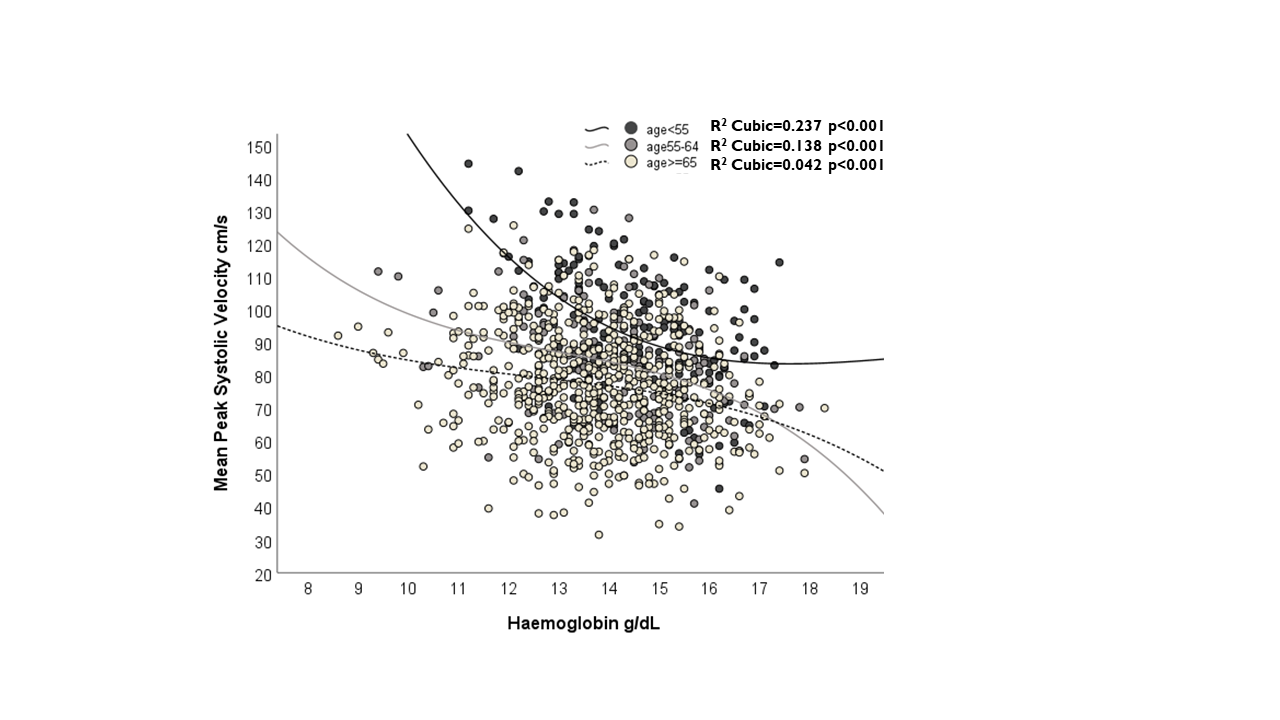


Supplementary Figure 2: lines of best fit on regression analysis between peak systolic velocity and haemoglobin in patients <55; 55-64 and ≥65 years of age.

**Detailed bibliography**

1. Henriksen OM, Gjedde A, Vang K, Law I, Aanerud J, Rostrup E. Regional and interindividual relationships between cerebral perfusion and oxygen metabolism. *J Appl Physiol* 2021; 130: 1836–1847.
2. Gur RC, Gur RE, Obrist WD, et al. Sex and handedness differences in cerebral blood flow during rest and cognitive activity. *Science* 1982; 217: 659-661.
3. Rodriguez G, Warkentin S, Risberg J, and Rosadini G. Sex Differences in Regional Cerebral Blood Flow. *JCBFM* 1988; 8: 783-789.
4. Aanerud J, Borghammer P, Rodell A, Jo´nsdottir KY, Gjedde A. Sex differences of human cortical blood flow and energy metabolism. *JCBF.* 2017; 37: 2433–2440.
5. Alisch JSR, Khattar N, Kim R, et al. Sex and age-related differences in cerebral blood flow investigated using pseudo-continuous arterial spin labelling magnetic resonance imaging. *Aging* 2021; 13: 4911-4925.
6. Tomoto T, Lu M, Khan AM, et al. Cerebral blood flow and cerebrovascular resistance across the adult lifespan: A multimodality approach. *JCBFM* 2023; 43: 962–976.
7. Tegeler CH, Crutchfield K, Katsnelson M, et al. Transcranial Doppler Velocities in a Large, Healthy Population. *J Neuroimaging* 2013; 23: 466-472.
8. Krejza J, Mariak Z, Walecki J, Szydlik P, Lewko J, Ustymowicz A. Transcranial Color Doppler Sonography of Basal Cerebral Arteries in 182 Healthy Subjects: Age and Sex variability and Normal Reference Values for Blood Flow Parameters *AJR.* 1999; 172: 213-218.
9. Vriens EM, Kraaier V, Musbach M, Wieneke GH, van Huffelen AC. Transcranial pulsed Doppler measurements of blood velocity in the middle cerebral artery: reference values at rest and during hyperventilation in healthy volunteers in relation to age and sex *Ultrasound Med Biol*. 1989; 15:1-8.
10. Bakker SLM, de Leeuw FE, den Heijer T, Koudstaal PJ, Hofman A, Breteler MMB. Cerebral haemodynamic in the elderly: the Rotterdam study. *Neuroepidemiology* 2004; 23: 178-184.
11. Messerli FH, Garavaglia GE, Schmieder RE, Sundgaard-Riise K, Nunez BD, Amodeo C. Disparate cardiovascular findings in men and women with essential hypertension. *Ann Intern Med* 1987; 107: 158-161.
12. Alfie J, Waisman GD, Galarza CR, et al. Relationship between systemic hemodynamics and ambulatory blood pressure level are sex dependent. *Hypertension* 1995; 26: 1195-1199.
13. Alonso-Nanclares L, Gonzalez-Soriano J, Rodriguez JR, DeFelipe J. Gender differences in human cortical synaptic density. *Proc Natl Acad Sci USA* 2008; 105: 1461–1469.
14. Yang D, Cabral D, Gaspard EN, Lipton RB, Rundek T, Derby CA. Cerebral Hemodynamics in the Elderly: A Transcranial Doppler Study in the Einstein Aging Study Cohort. *J Ultrasound Med* 2016; 35: 1907-1914.
15. Melamed E, Law S, Bentin S, Cooper G, Rinot Y. Reduction in Regional Cerebral Blood Flow During Normal Aging in Man. *Stroke* 1980; 11: 31-35.
16. Ibaraki M, Shinohara Y, Nakamura K, Miura S, Kinoshita F, and Kinoshita T. Interindividual variations of cerebral blood flow, oxygen delivery, and metabolism in relation to hemoglobin concentration measured by positron emission tomography in humans. *JCBFM* 2010; 30: 1296–1305.
17. von Kummer R, Scharf J, Back T, Reich H, Machens HG, Wildemann B. Autoregulatory Capacity and the Effect of Isovolemic Hemodilution on Local Cerebral Blood Flow. *Stroke*1988; 19: 594-597.
18. Kosinski PD, Croal PL, Leung J, et al. The severity of anaemia depletes cerebrovascular dilatory reserve in children with sickle cell disease: a quantitative magnetic resonance imaging study. *Br J Haematol* 2017; 176: 280–287.
19. Vorstrup S, Lass P, Waldemar G, et al. Increased cerebral blood flow in anaemic patients on long-term haemodialytic treatment. *J Cereb Blood Flow Metab* 1992; 12: 745-749.
20. Thomas DJ, Marshall J, Russell RW, et al. Effect of Haematocrit on cerebral blood flow in man. *Lancet* 1977; 310: 941-943.
21. Brown MM, Wade JP, Marshall J. Fundamental importance of arterial oxygen content in the regulation of cerebral blood flow in man. *Brain* 1985; 108: 81-93.
22. Brown MM, Marshall J. Regulation of cerebral blood flow in response to changes in blood viscosity. *Lancet* 1985; 1: 604-609.
23. Henriksen L, Paulson OB, Smith RJ. Cerebral blood flow following normovolemic hemodilution in patients with high hematocrit. *Ann Neurol* 1981; 9: 454–457.
24. Adams RJ, McKie VC, Hsu L, et al. Prevention of a first stroke by transfusions in children with sickle cell anemia and abnormal results on transcranial Doppler ultrasonography. *N Engl J Med* 1998; 339: 5–11.
25. Zierk J, Krebs A, Rauh M, et al. Blood count in adult and elderly individuals: defining the norm over eight decades of life. *Br J Haematol* 2020; 189: 777-789.
26. Vaccarino V, Abramson JL, Veledar E, Weintraub WS. Sex Differences in Hospital Mortality After Coronary Artery Bypass Surgery Evidence for a Higher Mortality in Younger Women. *Circulation* 2002; 105: 1176-1181.
27. Rothwell PM, Eliasziw M, Gutnikov SA, Warlow CP, HJM Barnett, for the Carotid Endarterectomy Trialists Collaboration. Endarterectomy for symptomatic carotid stenosis in relation to clinical subgroups and timing of surgery. *Lancet* 2004; 363: 915–24.
28. Ulug P, Sweeting MJ, von Allmen RS, Thompson SG, Powell JT, on behalf of the SWAN collaborators. Morphological suitability for endovascular repair, non-intervention rates, and operative mortality in women and men assessed for intact abdominal aortic aneurysm repair: systematic reviews with meta-analysis. *Lancet* 2017; 389: 2482–91.
29. Aaslid R, Lindegaard KF, Sorteberg W, Nornes H. Cerebral autoregulation dynamics in humans. *Stroke* 1989; 20: 45-52.
30. Kontos HA. Validity of Cerebral Arterial Blood Flow Calculations From Velocity Measurements. *Stroke* 1989; 20: 1-3.
31. Sorteberg W. Cerebral Artery Blood Flow Velocity and Cerebral Blood Flow. In: Newell DW, Aaslid R.*Transcranial Doppler.* Raven Press, Ltd., New York,1992.
32. Newell DW, Aaslid R, Lam A, Mayberg TS, Winn HR. Comparison of Flow and Velocity During Dynamic Autoregulation Testing in Humans. *Stroke* 1994; 25: 793-797.
33. Rothwell PM, Coull AJ, Silver LE, et al. Population-based study of event-rate, incidenc.e, case fatality, and mortality for all acute vascular events in all arterial territories (Oxford Vascular Study). *Lancet* 2005; 366: 1773–1783.
34. Mazzucco S, Li L, McGurgan IJ, Tuna MA, Brunelli N, Binney LE, Rothwell PM. Cerebral hemodynamic effects of early blood pressure lowering after TIA and stroke in patients with carotid stenosis. *Int J Stroke* 2022; 17: 1114-1120.
35. Schulz UGR, Rothwell PM. Major variation in carotid bifurcation anatomy: A possible risk factor for plaque development? *Stroke* 2001; 32: 2522-29.
36. Schulz UGR, Rothwell PM. Sex differences in carotid bifurcation anatomy and the distribution of atherosclerotic plaque. *Stroke* 2001; 32: 1525-1531.
37. Ford CS, Howard VJ, Howard G, Frye JL, Toole JF, McKinney WM. The sex difference in manifestations of carotid bifurcation disease. *Stroke* 1986; 17: 877-81.
38. Krejza J, Mariak Z, Huba M, Wolczynski S, Lewko J. Effect of endogenous oestrogen on blood flow through carotid arteries. *Stroke* 2001; 32: 30-36.
39. Miller KB, Howery AJ, River-Rivera LA, et al. Age-related reductions in cerebrovascular reactivity using 4D flow MRI. *Front Aging Neurosci* 2019; 11: 281.
40. Markus HS. Cerebrovascular abnormalities in Alzheimer’s dementia: a more tractable treatment target? *Brain* 2017; 140: 1822–1825.
41. Hogue CW Jr, Barzilai B, Pieper KS, Coombs LP, DeLong ER, Kouchoukos NT, Dávila-Román VG. Sex Differences in Neurological Outcomes and Mortality After Cardiac Surgery. A Society of Thoracic Surgery National Database Report. *Circulation* 2001; 103: 2133-2137.
42. Butcher A, Richards T, Stanoth SJ, Klein AA. Diagnostic criteria for pre-operative anaemia. Time to end sex discrimination. *Anaesthesia* 2017; 72: 811-814.
43. Gagnon DR, Zhang TJ, Brand FN, Kannel WB. Hematocrit and the risk of cardiovascular disease—The Framingham Study: A 34-year follow-up. *AHJ* 1994; 127: 674-682.
44. Wolters FJ, Zonneveld HI, Licher S, et al. Hemoglobin and anemia in relation to dementia risk and accompanying changes on brain MRI. *Neurology* 2019; 93: e917-e926.
45. Wang H, O’Reilly EJ, Weisskopf MG,et al. Smoking and Risk of Amyotrophic Lateral Sclerosis. A Pooled Analysis of 5 Prospective Cohorts. *Arch Neurol* 2011; 68: 207-213.
46. Widdowson EM, McCance RA. Iron in human nutrition. *J Hyg* 1936; 36:13–23.
47. Weyand AC, McGann PT, Sholzberg M. Sex specific definitions of anaemia contribute to health inequity and sociomedical injustice. *Lancet Haematol* 2022; 9: e6-e8.
48. WHO. The urgent need to implement patient blood management: policy brief. Accessed 4/1/2024. <https://www.who.int/publications/i/item/9789240035744>.
